# Open-Ring Butenolides from a Marine-Derived Anti-Neuroinflammatory Fungus *Aspergillus terreus* Y10

**DOI:** 10.3390/md16110428

**Published:** 2018-11-02

**Authors:** Long-He Yang, Han Ou-Yang, Xia Yan, Bo-Wen Tang, Mei-Juan Fang, Zhen Wu, Jing-Wei Chen, Ying-Kun Qiu

**Affiliations:** 1 Engineering Research Center of Marine Biological Resource Comprehensive Utilization, Third Institute of Oceanography, State Oceanic Administration, Xiamen 361005, China; longheyang@tio.org.cn; 2 Fujian Provincial Key Laboratory of Innovative Drug Target Research, School of Pharmaceutical Sciences, Xiamen University, South Xiang-An Road, Xiamen 361102, China; tbwxmu@gmail.com (B.-W.T.); fangmj@xmu.edu.cn (M.-J.F.); wuzhen@xmu.edu.cn (Z.W.); 3 Institute of Drug Discovery Technology, Ningbo University, Ningbo 315832, China; raul07cf@163.com (H.O.-Y.); yanxia@nbu.edu.cn (X.Y.); 4 Key Laboratory for Chemical Biology of Fujian Province, College of Chemistry and Chemical Engineering, Xiamen University, Xiamen 361005, China; chembio@xmu.edu.cn

**Keywords:** *Aspergillus terreus* Y10, asperteretal F, G_1_, G_2_, H, open-ring butenolide, microglial, anti-neuroinflammatory activity

## Abstract

To investigate structurally novel and anti-neuroinflammatory natural compounds from marine-derived microorganisms, the secondary metabolites of *Aspergillus terreus* Y10, a fungus separated from the sediment of the coast in the South China Sea, were studied. Three new compounds (**2**–**4**), with novel open-ring butenolide skeletons, were isolated from the ethyl acetate extract of the culture medium. In addition, a typical new butenolide, asperteretal F (**1**), was found to dose-dependently inhibit tumor necrosis factor (TNF-α) generation with an IC_50_ of 7.6 μg/mL. The present study shows the existence of open-ring butenolides, and suggests that butenolides such as asperteretal F (**1**) are a promising new anti-neuroinflammatroy candidate for neurodegenerative diseases.

## 1. Introduction

Fungi are a large source of bioactive metabolites [1]. *Aspergillus* is a large genus containing 180 species of fungi [2,3]. *Aspergillus terreus* has been isolated from terrestrial and marine sources. Butenolides with a basal skeleton of a five-membered lactone bearing two aromatic rings, are important bioactive metabolites of *A. terreus* [4,5]. It has been reported that butenolide compounds such as aperteretal A–C [6] and asperteretal D–F [7] exhibited a wide range of activities, such as antimicrobial, cytotoxic activities, α-glucosidase inhibitory activities and anti-inflammatory activities [6,8,9,10,11].

Microglia cells are one of the important immune cells in the central nervous system (CNS). They usually play the role of immune surveillance under a resting state. Under normal circumstances, such cells can be activated quickly to eliminate pathological insults. However, in some cases, the continuous activation of microglia cells excrete a variety of inflammatory substances, such as tumor necrosis factor (TNF-α) and interleukin 1-β (IL1-β), leading to chronic inflammation of the central nervous system [12,13,14]. It is believed that several neurodegenerative diseases, such as Alzheimer′s disease (AD), Parkinson′s disease (PD), multiple sclerosis and human immunodeficiency virus (HIV)-associated dementia are related to the excessive and uncontrolled activation of microglia cells [15,16,17,18]. Therefore, use of small molecules to modulate the uncontrolled microglia cells is an important strategy in therapy for this kind of disease.

**Figure 1:**
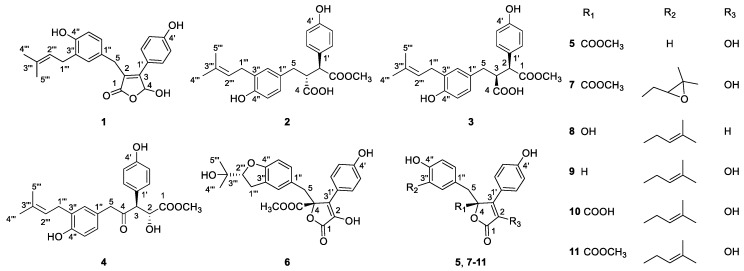
Structures of compounds **1**–**11** isolated from an extract of *Aspergillus terreus* Y10.

In this study, three new compounds (**2**–**4**), with novel open-ring butenolide skeletons, were isolated from the ethyl acetate extract of *Aspergillus terreus* Y10, a fungus separated from the sediment of the coast in the South China Sea. In addition, a typical new butenolide, asperteretal F (**1**), together with 7 known butenolide derivatives (**5**–**11**), were also isolated (Figure 1). The anti-neuroinflammatory activity of these compounds were also evaluated in BV2 microglia cells. The new butenolide, asperteretal F (**1**) was found to dose-dependently inhibit the TNF-α generation with an IC_50_ of 7.6 μg/mL.

## 2. Results

### 2.1. Structural Identification of New Compounds

**Figure 2:**
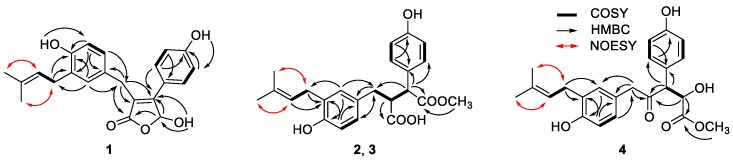
Key ^1^H–^1^H correlation spectroscopy (COSY), heteronuclear multiple-bond correlation spectroscopy (HMBC), and nuclear Overhauser effect spectroscopy (NOESY) correlations of **1**–**4**.

Compound **1** (asperteretal F) was isolated as colorless oil. The molecular formula of C_22_H_22_O_5_, which gave 12 unsaturation degrees, was established by the positive and negative high-resolution electrospray ionisation mass spectrometry (HR-ESI-MS) ion peak at *m*/*z* 389.1354 [M + Na]^+^ (calcd for C_22_H_22_O_5_Na, 389.1359), and 365.1400 [M − H]^−^ (calcd. for C_22_H_21_O_5_, 365.1400), respectively. The ultraviolet (UV) maximum absorption wavelength at *λ*_max_ (log*ε*): 305 (3.45) nm indicating the presence of conjugated unsaturated lactone carbonyl. The infrared (IR) spectrum of **1** indicated the presence of a conjugated γ-lactone carbonyl signal at 1727 cm^−1^. The ^1^H and ^13^C nuclear magnetic resonance (NMR) spectra (Table 1), including the spectrum of distortionless enhancement by polarization transfer (DEPT), clearly showed a carbonyl carbon and 16 olefinic carbons in the *sp*^2^ low-field region, attributed to a *para*-hydroxyl substituted benzene ring, a 3,4-disubstituted benzyl, and two ethylenic double bonds. The *sp*^3^ high-field region showed the existence of two methyl and two methylenes. In addition, a methine bearing to 2 oxygen atoms was found in the low field of the *sp*^3^ region at *δ*_H_ 6.53 (br. d, *J* = 7.3 Hz) and δ_C_ 97.7. All these spectroscopic data were similar to those of a known compound, asperteretal D [7], expect for the absence of a methoxyl on C-4, which was confirmed by the high-field shifting of C-4 from δ_C_ 102.6 in asperteretal D to 97.7. In addition, the heteronuclear multiple-bond correlation spectroscopy (HMBC) correlations from H-5 to C-1, C-2 and C-3, and from H-6″ and H-2″ to C-5 implied that the 4′-hydroxy-3′-isopentenyl benzyl moiety located at C-2 position. Comprehensive heteronuclear single quantum coherence spectroscopy (HSQC), ^1^H–^1^H correlation spectroscopy (COSY), HMBC and nuclear Overhauser effect spectroscopy (NOESY) analysis allowed the complete assignment of the proton and carbon signals for **1** (Table 1 and Figure 2). As a result, the structure of **1** was elucidated as shown in Figure 1, named asperteretal F.

**Figure 3:**
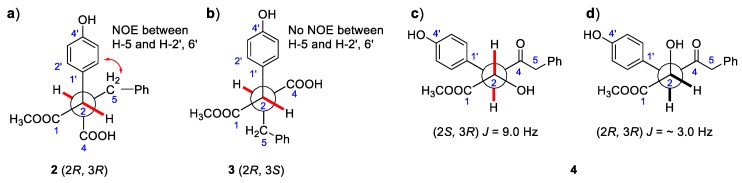
Key ^1^H-^1^H coupling constants and NOESY correlations of **2**–**4**.

**Figure 4:**
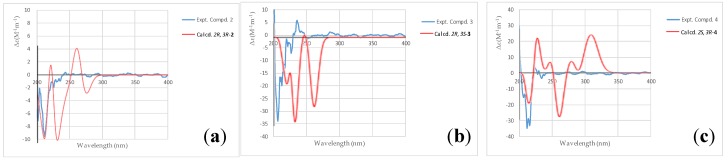
Calculated and experimental electronic circular dichroism (ECD) spectra of the compounds **2**–**4**. (a) Calculated (*2R*, *3R*) and experimental ECD of compound **2**; (b) Calculated (*2R*, *3S*) and experimental ECD of compound **3**; (c) Calculated (*2S*, *3R*) and experimental ECD of compound **4**.

Compound **2** (asperteretal G_1_) was also obtained as a white powder. The molecular formula of C_23_H_24_O_6_, giving 11 unsaturation degrees, was derived by the positive HR-ESI-MS ion peak of [M + Na]^+^ at *m/z* 421.1622 (calcd for C_23_H_25_O_6_, 421.1622), and negative ion peak of 397.1661 [M − H]^−^ (calcd. for C_22_H_21_O_5_, 397.1662), respectively. The IR spectrum (KBr) showed the presence of an associated carbonyl signal at 1716 cm^−1^. The *para*-hydroxyl substituted benzene ring, the 3,4-disubstituted benzyl, and the isopentenyl side chain signals could also be found in the NMR spectra of **2**. However, as compared with those data in **1**, the ^1^H-NMR chemical shifts of H-2′, 6′, H-2″, and H-6″ were obviously high-field shifted from *δ*_H_ 7.45 to *δ*_H_ 7.18 (H-2′, 6′), from *δ*_H_ 6.88 to *δ*_H_ 6.63 (H-2″), and from *δ*_H_ 6.82 to *δ*_H_ 6.65 (H-6″), respectively. The evidence indicates that the five-membered conjugated lactone ring could be absent in **2**, resulting in the dismission of an magnetically anisotropic effect. Moreover, the ^13^C-NMR spectra data of **2** differed from those of **1** around C-1, C-2, C-3 and C-4. Two carbonyl signals at *δ*_C_ 173.3 (C-1) and 175.5 (C-4) were found in the low field of the ^13^C NMR. In addition, the ^1^H-NMR signals at *δ*_H_ 3.62 (1H, d, *J* = 11.0 Hz, H-2) and 3.23 (1H, m, H-3), as well as their corresponding ^13^C-NMR and DEPT signals at *δ*_C_ 52.1 (C-2) and 49.4 (C-3), were attributed to two methines on the aids of HSQC spectra. The ^1^H-^1^H COSY correlations between H-2 (*δ*_H_ 3.62) and H-3 (*δ*_H_ 3.23), and between H-3 and H-5 [*δ*_H_ 2.63 (1H, dd, *J* = 13.9, 3.9 Hz) and 2.37 (1H, dd, *J* = 13.9, 7.9 Hz)] revealed the linkage of -CH-CH-CH_2_- fragment from C-2, C-3 to C-5. In the HMBC spectrum, a methoxyl signal *δ*_H_ 3.58 (3H, s) exhibited correlation with one of the carbonyl *δ*_C_ 173.3 (C-1), indicating the presence of -COOCH_3_. The key HMBC correlations from H-2 to C-1, C-1′ and from H-3 to C-4 allowed the elucidation of the structure of **2**. The relative configuration between C-2 and C-3 was revealed by the NOESY spectrum. In detail, the coupling constant between H-2 and H-3 was 11.6 Hz, indicating the trans-coplanar positional relationship between the two C-H bonds. The NOESY correlation between H-5 and H-2′, 6′ in the NOESY spectrum revealed the relative configuration of **2**, as shown in Figure 3a. The theoretical electronic circular dichroism (ECD) spectra of *2R*, *3R*-2 and *2S*, *3S*-2 were further calculated and compared with the experimental ones to determine the absolute configurations. As shown in Figure 4a, the experimental ECD spectrum was similar to the calculated ECD spectrum of *2R*, *3R*-**2** and the absolute configuration of **2** was determined as *2R*, *3R*.

Compound **3** (asperteretal G_2_) was isolated as a white solid. Its molecular formula of C_23_H_24_O_6_ was induced by the HR-ESI-MS ion peak at *m/z* 421.1621 [M + Na]^+^ (calcd for C_23_H_25_O_6_, 421.1622), and at *m/z* 397.1661 [M − H]^–^ (calcd. for C_22_H_21_O_5_, 397.1662). The IR spectrum (KBr) also showed an associated carbonyl signal at 1719 cm^−1^. Both 1D-NMR and 2D-NMR are very similar to **2**, except for the ^1^H and ^13^C-NMR signals around C-2 and C-3. Most of the HSQC, ^1^H-^1^H COSY, HMBC correlations of **3** were similar to those of **2**, indicating that **3** was the epimer of **2**. The relative configuration of **3** was different from that of **2** in C-2 and C-3, which was also elucidated by the NOESY spectrum. The coupling constant between H-2 and H-3 of **3** was 11.0 Hz, indicating that the two C–H bonds were also in trans-coplanar position. The NOESY correlation between H-5 and H-2′, 6′ was not found in the NOESY spectrum of **3**, revealing that the relative configuration of **3** was different with that of **2** (Figure 3b). As shown in Figure 4b, the experimental ECD spectrum of **3** was close to the calculated ECD spectrum of *2R*, *3S*-**3** and the absolute configuration of **3** was determined as *2R*, *3S*.

Compound **4** (asperteretal H) was isolated also as a white solid. The molecular formula of C_23_H_26_O_6_, giving 11 unsaturation degrees, was established by the HR-ESI-MS ion peak at *m/z* 421.1621 [M + Na]^+^ (calcd. for C_24_H_32_O_4_Na, 421.1622). A keto IR signal emerged at 1740 cm^−1^.The ^1^H, ^13^C NMR and DEPT signals attributed to the two benzene rings and the isopentenyl side chain were closed to those of **1**, **2**, and **3**. In the low field of ^13^C NMR of **3**, a ketone carbonyl bearing to *sp*^3^ carbons could be found at *δ*_C_ 207.6. In the HMBC spectrum, correlations between this ketone carbonyl and H-5 (*δ*_H_ 3.49 (2H, s)), H-3 (*δ*_H_ 4.06, (1H, d, *J* = 9.2 Hz)) and H-2 (*δ*_H_ 4.34, (1H, t, *J* = 7.3 Hz)) could be found, revealing their linkage. The ^1^H signal belonging to a methoxyl at *δ*_H_ 3.56 (3H, s) showed correlation with another carbonyl at *δ*_C_ 173.4 in the HMBC spectrum. This -COOCH_3_ group was connected to C-2, revealed by the HMBC correlation from H-2 to C-1. Thus, the structure of **4** was elucidated as shown in Figure 2. The relative configurations of C-2 and C-3 were revealed by the coupling constant between H-2 and H-3 (*J*_H-2,3_ = 9.0 Hz) and based on its preferential conformation (Figure 3c). With the aid of calculated and experimental ECD spectra, the absolute configurations of C-2 and C-3 were determined as *2S* and *3R*, respectively (Figure 4c). 

The structures of compounds **5**–**11** were elucidated by the comparison of their MS and NMR data with those reported in literature, and they were identified as: butyrolactone II (**5**) [19], butyrolactone IV (**6**) [10], butyrolactone III (**7**) [20], butyrolactone IX (**8**) [21], 3-hydroxy-5-[[4-hydroxy-3-(3-methyl-2-buten-1-yl) phenyl] methyl]-4-(4-hydroxyphenyl)-(5*H*)-furan-one (**9**) [6], 5-dihydro-4-hydroxy-2-[[4-hydroxy-3-(3-methyl-2-butenyl) phenyl] methyl]-3-(4-hydroxyphenyl)-5-oxo-furancarboxylic acid) (**10**) [22], butyrolactone I (**11**) [23]. Their ^1^H and ^13^C NMR were provided in Tables S1 and S2 in the Supplementary Materials.

### 2.2. Cytotoxicity

**Figure 5:**
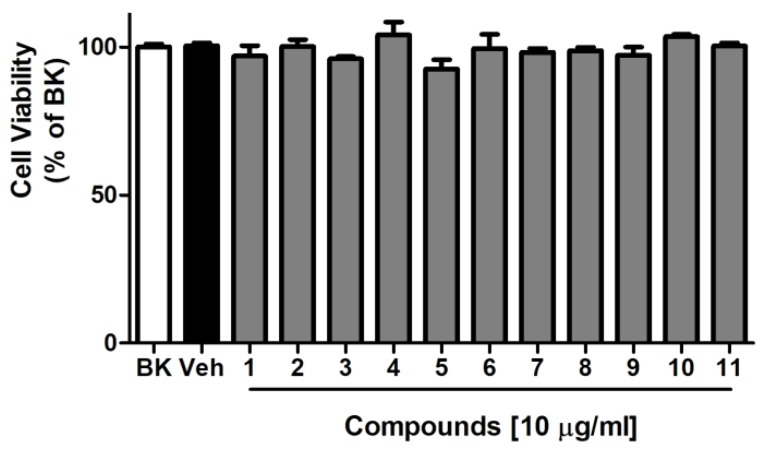
Compounds have no cytotoxicity on the viability of BV-2 cells at concentrations of 10 μg/mL at 37 °C for 48 h. Cytotoxicity was assessed by cell counting kit-8 (CCK-8) assay. Values are expressed as mean ± standard deviation (SD), *n* = 3.

The cytotoxicity of compounds **1**–**11** in BV-2 cells were tested by using the cell counting kit-8 (CCK-8) assay kit. The result showed that compounds **1**–**11** did not exhibit an obvious cytotoxic effect at the employed concentrations (10 µg/mL) (Figure 5).

### 2.3. Inhibitory Effect of Compounds on Lipopolysaccharide (LPS)-Induced Tumor Necrosis Factor (TNF-α) Generation

**Figure 6:**
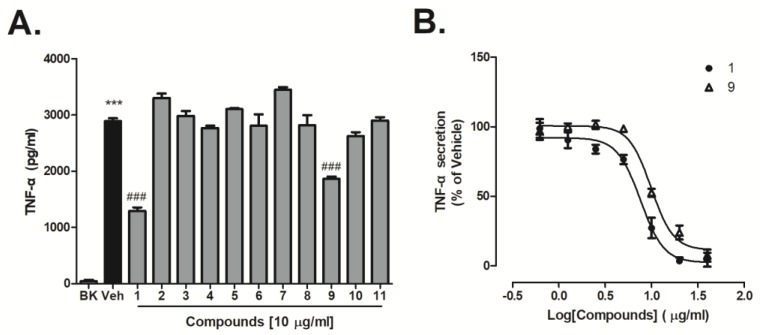
Effect of compounds **1**–**11** on lipopolysaccharide (LPS)-induced tumor necrosis factor (TNF-α) secretion in BV-2 cells. Cells were pre-incubated with vehicle (DMSO, 0.1%) or compounds (**A**) (**1**–**11**, 10 μg/mL) and (**B**) (**1** and **9**, 0.6–40 μg/mL) for 1 h before stimulation with 1 μg/mL of LPS for 6 hours. TNF-α production was measured by enzyme-linked immunosorbent assay (ELISA). Data represent mean ± SEM measurements in triplicates. Data were analyzed by a one-way analysis of variance (ANOVA) followed by a Tukey′s multiple comparison test. *** *p* < 0.001 vs. BK; ### *p* < 0.001 vs vehicle.

The effects of these compounds on BV-2 microglia activation was further investigated. Cells were pre-incubated with compounds **1**–**11** (10 µg/mL) for an hour then activated by lipopolysaccharide (LPS) for 6 hours. TNF-α production was measured as a marker of cell activation. In this setting, compounds **1** and **9** inhibited TNF-α production by 55.1% and 35.5% at a dose of 10 μg/mL (Figure 6A). The inhibitory effect of compounds **1** and **9** at different concentration on LPS-activated BV-2 cells was further evaluated. Results showed that both compounds **1** and **9** dose-dependently decreased LPS-induced TNF-α generation (Figure 6B). IC_50_ values were determined for compound **1** (IC_50_: 7.6 ± 1.1 μg/mL) and compound **9** (IC_50_: 9.9 ± 1.06 μg/mL) at concentrations ranging from 0.6 to 40 μg/mL (Figure 6B).

## 3. Discussion

Butenolides with a basal skeleton of a five-membered lactone bearing two aromatic rings, are important bioactive metabolites of *A. terreus*. In this study, the open-ring butenolides were isolated for the first time. These new compounds may contribute to revealing the biosynthesis pathways of butenolides. 

Unlike target-based approaches, phenotypic-based compound screening is considered to play an efficient role in early drug discovery, especially when the molecular basis of a disease is not clearly understood. Proinflammatory cytokines, such as TNF-α, IL-6, which are released from activated microglia are considered to play important roles in the pathogenesis of neuro-inflammation in the CNS [6]. These cytokines can be used as hallmarks in phenotypic assays during the early stages of microglia activation. In this study, four new compounds, together with 7 known butenolide derivatives were isolated from the ethyl acetate extract of marine-derived *Aspergillus terreus* Y10. The effects of the compounds against TNF-α generation was evaluated in LPS-induced BV-2 cells. At 10 μM, we found only compounds **1** and **9** inhibited expression of TNF-α in LPS-activated microglia. The opened-ring butenolides (**2**–**4**) did not show potent inhibitory activity. Further research found that compounds **1** and **9** dose-dependently reduced TNF-α procution in LPS-activated microglia cells with IC_50_ of 7.6 and 9.9 μg/mL, respectively. The activity difference among these butenolides may be related to substituent in the C-5 position, because large groups (COOH, COOCH_3_, and OH) around C-5 greatly reduced the anti-inflammatory activity. Although the anti-inflammatory activity of butenolides has been reported, it is the first time their activity on neuro microglia cells has been discussed.

Taken together, the present study shows the existence of open-ring butenolides. The potential anti-neuroinflammatory activity of asperteretal F (**1**) in LPS-induced microglia cells was also revealed. Our findings suggest that asperteretal F (**1**) is a promising new anti-neuroinflammatroy candidate for neurodegenerative diseases, although the mechanism of action should be further clarified.

## 4. Materials and Methods 

### 4.1. General Experimental Procedures

Silica gel (Yantai Chemical Industry Research Institute, Yantai, China) and Cosmosil 75 C_18_-MSII (75 μm, Nakalai Tesque Co. Ltd., Kyoto, Japan) were used in open-column chromatographic separation. A Shimadzu LC-20AP preparative high-performance liquid chromatography (HPLC) system (Shimadzu corporation, Tokyo, Japan), including a quaternary gradient solvent delivery unit LC-20A, a photodiode array detector (SPD-M20A) and a fraction collector (Shimadzu FRC-10A) was used in the preparative separation, via a preparative Cosmosil ODS column (250 mm × 20.0 mm i.d., 5 μm, Cosmosil, Nakalai Tesque Co. Ltd., Kyoto, Japan). UV spectra were recorded on a Shimadzu UV-260 spectrometer (Shimadzu Corporation, Tokyo, Japan). IR spectra were determined on a Perkin-Elmer 683 infrared spectrometer (PerkinElmer, Inc., Waltham, MA, USA) in KBr pellets. Optical rotations were measured using a JASCO P-200 polarimeter (JASCO Corporation, Tokyo, Japan) with a 5-cm cell. Thermo Q-Exactive Mass spectrometer (Thermo Fisher Scientific Corporation, Waltham, MA, USA) equipped with electrospray ionization source (ESI) was used to obtain the HR-ESI-MS spectra. The 1D NMR and 2D NMR spectra were were taken on a Brucker Avance III 600 FT NMR spectrometer (Bruker Corporation, Billerica, MA, USA), with tetramethylsilane (TMS) as the internal standard. The circular dichroism (CD) spectra were acquired on a Chirascan circular dichroism spectrometer (Applied Photophysics Ltd., Leatherhead, UK). 

### 4.2. Eletronic Circular Dichroism (ECD) Calculations

The theoretical eletronic circular dichroism (ECD) spectra of the isolated compounds were calculated on the basis of the relative configurations determined by their NOESY spectra and *J* value in ^1^H NMR. Conformational analyses and density functional theory (DFT) calculations were used to generate and optimize the conformers with energy. The ECD calculations were performed as the method descripted in a reported article [24].

### 4.3. Fungal Strain and Fermentation

The fungal strain *Aspergillus terreus* was isolated from a sediment sample collected in the coastal area of Hainan, China. The strain was identified based on internal transcribed spacer (ITS) region sequence analysis of its rDNA. The strain is stored at the Engineering Research Centre of Marine Biological Resource Comprehensive Utilization, Third Institute of Oceanography, State Oceanic Administration.

The fungus was grown in Erlenmeyer flasks (60 × 500 mL) containing 1% glucose, 2% maltose, 0.1% corn steep liquor, 0.3% yeast extract, 1% monosodium glutamate, 2% mannitol, 0.05% KH_2_PO_4_, 0.03% MgSO_4_·7H_2_O in natural seawater at pH 7.5. The fungus was grown in stationary culture at 22 °C for 30 days. 

### 4.4. Extraction and Isolation

The medium of *A. terreus* Y10 (15 L) was extracted with ethyl acetate (15 L, *v:v* = 1:1) three times and concentrated under reduced pressure at 40 °C to afford 12.0 g residue. The residue was divided into 9 fractions (Fr. 1~9) over silica gel column eluted with petroleum ether-ethyl acetate (*v*/*v*) (20:1; 10:1; 5:1; 2:1; 1:1) and chloroform-methyl alcohol (*v*/*v*) (50:1; 20:1; 10:1; 5:1). The most active and most weighty fraction, Fr. 5 (6.2 g) was subjected to octadecylsilyl (ODS) chromatography and eluted with CH_3_OH–H_2_O (20–100%) to give 9 subfractions. Then Fr. 5.4 was purified by preparative reversed-phase HPLC using a C_18_ column and isocratic eluted with acetonitrile-H_2_O (35:65) to obtain compound **5** (65.7 mg). Fr. 5.6 was purified by preparative reversed-phase HPLC isocratic eluted with methanol-H_2_O (55:45) to obtain compound **6** (96.0 mg), compound **7** (33.4 mg) and compound **8** (17.2 mg). Fr. 5.7 was also purified by preparative HPLC isocratic eluted with acetonitrile-H_2_O (40:60) to obtain compound **1** (5.5 mg), **2** (7.0 mg), **3** (14.3 mg), **4** (4.5 mg), **9** (91.6 mg), **10** (4.3 mg) and **11** (196.0 mg).

Asperteretal F (**1**): colorless oil; [α]${}_{D}^{25}$ −126° (*c* = 0.1, CH_3_OH), IR (KBr) (ν_max_): 3419, 1726, 1584 cm^−1^. UV (CH_3_OH) λ_max_ (log *ε*): 202 (4.00) nm, 305 (3.45) nm. ^13^C NMR (125 MHz, DMSO-*d_6_*) and ^1^H NMR (600 MHz, DMSO-*d_6_*) spectral data were listed in Table 1; HR-ESI-MS: *m*/*z* 389.1354 (calcd. for C_22_H_22_O_5_Na, 389.1359) in positive mode, and *m*/*z* 365.1400 (calcd. for C_22_H_21_O_5_, 365.1400) in negative mode.

Asperteretal G_1_ (**2**): white powder; [α]${}_{D}^{25}$ −16° (*c* = 0.1, CH_3_OH), IR (KBr) (ν_max_): 3421, 1716, 1568, 1451 cm^−1^. UV (CH_3_OH) λ_max_ (log *ε*): 203 (4.38) nm, 221 (3.89) nm, 231 (3.94) nm. ^13^C NMR (125 MHz, DMSO-*d_6_*) and ^1^H NMR (600 MHz, DMSO-*d_6_*) spectral data were listed in Table 1; HR-ESI-MS: *m*/*z* 421.1622 (calcd. for C_23_H_26_O_6_Na, 421.1622) in positive mode, and *m*/*z* 397.1661 (calcd. for C_23_H_25_O_6_, 397.1662) in negative mode.

Asperteretal G_2_ (**3**): white powder; [α]${}_{D}^{25}$ 2° (*c* = 0.1, CH_3_OH), IR (KBr) (ν_max_): 3423, 1719, 1568, 1447, 1264 cm^−1^. UV (CH_3_OH) λ_max_ (log *ε*): 203 (4.37) nm, 221 (3.88) nm, 231 (3.94) nm. ^13^C NMR (125 MHz, DMSO-*d_6_*) and ^1^H NMR (600 MHz, DMSO-*d_6_*) spectral data were listed in Table 1; HR-ESI-MS: *m*/*z* 421.1621 (calcd. for C_23_H_26_O_6_Na, 421.1622) in positive mode, and *m*/*z* 397.1659 (calcd. for C_23_H_25_O_6_, 397.1662) in negative mode.

Asperteretal H (**4**): white powder; [α]${}_{D}^{25}$ 40° (*c* = 0.1, CH_3_OH), IR (KBr) (ν_max_): 3396, 1740 cm^−1^. UV (CH_3_OH) λ_max_ (log *ε*): 202 (4.07) nm, 280 (2.82) nm. ^13^C NMR (125 MHz, DMSO-*d_6_*) and ^1^H NMR (600 MHz, DMSO-*d_6_*) spectral data were listed in Table 1; HR-ESI-MS: *m*/*z* 421.1621 (calcd. for C_23_H_26_O_6_Na, 421.1622) in positive mode.

### 4.5. Cell Cultivation

The murine microglia cell line BV-2 was purchased from FuHeng BioLogy (Shanghai, China). These cells were maintained in Dulbecco′s modified Eagle′s medium (DMEM, Hyclone, Logan, UT, USA) supplemented with 1% penicillin-streptomycin (100 U/mL penicillin and 100 μg/mL streptomycin) and 10% of fetal bovine serum (PAN Biotech, Aidenbach, Germany). The cells were incubated in a humidified atmosphere of 5% CO_2_ at 37 °C.

### 4.6. CCK-8 Cytotoxicity Assay

Cell viability of compounds **1**–**11** were assessed in microglia cell line using the cell counting kit-8 (CCK-8, Dojindo, Japan) according to the manufacturer′s instructions. Briefly, BV-2 cells (1 × 10^4^ cells/well) were plated in 96-well plates. The other day, cells were cultured in fresh medium containing test samples (10 μg/mL in 1% DMSO) for 48 h. After 48 h incubation, CCK-8 (10 μl) solution was added to the wells and incubated for 2 h. The optical density of the solution was measured with at 450 nm with a microplate reader (SpectraMax Plus^384^, Molecular Device, San Jose, CA, USA). The optical density of control cells (BK) was taken as 100% viability.

### 4.7. TNF-α Quantification by Enzyme-Linked Immunosorbent Assay (ELISA)

Compounds were pretreated for 1 h followed by 1 μg/mL LPS, activated for 6 hours in BV-2 cells, then the cell culture supernatants were collected. TNF-α producted in the medium was determined by Valukine^TM^ ELISA Kit (R & D Systems, Shanghai, China) according to the manufacturer′s instructions.

## Figures and Tables

**Table 1:** ^1^H, ^13^C nuclear magnetic resonance (NMR) data of compounds **1–4**.

Position	^1^H-NMR [δ_H_ (*J* in Hz)]				^1^C-NMR [δ_C_]			
	1	2	3	4	1	2	3	4
1					172.9	173.9	173.3	173.4
2		3.59 d (11.6)	3.61 d (11.0)	4.34 dd (9.0, 7.3)	124.3	52.1	53.3	71.9
3		3.28 m	3.13 m	4.06, d (9.2)	156.6	49.4	51.8	59.9
4	6.53 br. d (7.3)	12.40 br. s [COOH]	12.38 br. s [COOH]		97.7	175.5	174.1	207.6
5	3.70 d (15.2) & 3.59 d (15.4)	2.62 dd (13.9, 3.9) & 2.37, dd (13.9, 7.9)	2.67 dd (13.4, 9.9) & 2.63 (13.4, 3.5)	3.50 d (16.3) &3.47 d (16.5)	29.0	34.3	37.1	47.4
1′					122.1	127.4	127.5	125.6
2′, 6′	7.45 d (8.1)	7.18 d (8.4)	7.08 d (8.4)	7.00, d (8.4)	130.7	130.1	129.8	130.9
3′, 5′	6.84 d (7.9)	6.84 d (8.3)	6.67 d (8.3)	6.72, d (8.4)	116.0	116.1	115.6	115.8
4′					159.7	157.4	157.3	157.2
1″					127.9	128.3	129.1	124.9
2″	6.88 br. s	6.63 br. s	6.80 d (1.7)	6.58 br. s	129.5	130.3	130.1	130.9
3″					128.1	126.7	127.1	127.5
4″					153.8	153.8	153.8	154.0
5″	6.68 d (8.1)	6.67 d (8.1)	6.65 d (7.7)	6.65 d (8.1)	115.3	114.9	115.1	115.0
6″	6.82 br. d (9.0)	6.65 dd (8.1,1.7)	6.71, dd (8.1,1.7)	6.59 dd (8.1, 1.8)	126.3	127.3	127.3	128.2
1‴	3.15, 2H, br. d (7.3)	3.21 dd (15.8, 8.1) & 3.17 dd (15.8, 7.9)	3.16, 2H, br. d (7.2)	3.13, 2H, br. d (7.3)	28.4	28.4	28.6	28.4
2‴	5.20 br. t (7.3)	5.28 br. t (7.1)	5.25 br. t (7.3)	5.20 br. t (7.3)	123.2	123.3	123.4	123.3
3‴					131.8	131.6	131.5	131.6
4‴	1.66, 3H, s	1.76, 3H, s	1.69, 3H, s	1.67, 3H, s	26.0	26.0	26.0	26.0
5‴	1.62, 3H, s	1.71, 3H, s	1.67, 3H, s	1.64, 3H, s	18.0	18.1	18.1	18.1
COOCH_3_		3.53, 3H, s	3.58, 3H, s	3.56, 3H, s		51.3	52.4	52.0
2/4-OH	7.80 br. d (8.1) [4-OH]			5.70, d (7.2) [2-OH]				
4′-OH	10.09 br. s	9.59 br. s	9.50 br. s	9.41, br.s				
4″-OH	9.18 br. s	9.19 br.s	9.16 br.s	9.18, br.s				

## Supplementary Materials

The following are available online at http://www.mdpi.com/1660-3397/16/11/428/s1, Table S1: ^1^H NMR data of compounds **5**–**11**; Table S2: ^13^C NMR data of compounds **5**–**11**.
