# Supplementary material for: Open-Ring Butenolides from a Marine-Derived Anti-Neuroinflammatory Fungus Aspergillus terreus Y10
Source: Mar Drugs. 2018 Nov 2;16(11):428. doi: 10.3390/md16110428 (PMC6267272; doi:10.3390/md16110428)
Supplement: Supplementary file 1 [file marinedrugs-16-00428-s001.pdf]

Open-Ring Butenolides from a Marine-Derived Anti-Neuroinflammatory Fungus *Aspergillus Terreus* Y10

Content

Content	Page
**Table S1.** ^{1}H-NMR data (600 MHz, DMSO-d_6) of compounds **5-11**	2
**Table S2.** ^{13}C-NMR Data of compounds **5-11** (DMSO-d_6, 125 MHz)	3
Figure S 1. Infrared (IR) spectrum **of** compound **1**	4
Figure S 2. High-resolution electrospray ionisation mass **spectrometry** (HR-ESI-MS) spectra of	5
Figure S 3. $^{1H-NMR}$ (600 MHz, DMSO-d6) of compound **1**	6
Figure S 4. $^{13C-NMR}$ (150 MHz, DMSO-d6) of compound **1**	7
Figure S 5. DEPT of compound **1**	8
Figure S 6. Heteronuclear single quantum **coherence** spectroscopy (HSQC) of compound **1**	9
Figure S 7. $^{1H-1H}$ COSY of compound **1**	10
Figure S 8. HMBC of compound **1**	11
Figure S 9. NOESY of compound **1**	12
Figure S 10. IR spectrum of compound **2**	13
Figure S 11. HR-ESI-MS spectra of compound **2**	14
Figure S 12. $^{1H-NMR}$ (600 MHz, DMSO-d6) of compound **2**	15
Figure S 13. $^{13C-NMR}$ (150 MHz, DMSO-d6) of compound **2**	16
Figure S 14. DEPT of compound **2**	17
Figure S 15. HSQC of compound **2**	18
Figure S 16. $^{1H-1H}$ COSY of compound **2**	19
Figure S 17. HMBC of compound **2**	20
Figure S 18. NOESY of compound **2**	21
Figure S 19. IR spectrum of compound **3**	22
Figure S 20. HR-ESI-MS spectra of compound **3**	23
Figure S 21. $^{1H-NMR}$ (600 MHz, DMSO-d6) of compound **3**	24
Figure S 22. $^{13C-NMR}$ (150 MHz, DMSO-d6) of compound **3**	25
Figure S 23. DEPT of compound **3**	26
Figure S 24. HSQC of compound **3**	27
Figure S 25. $^{1H-1H}$ COSY of compound **3**	28
Figure S 26. HMBC of compound **3**	29
Figure S 27. NOESY of compound **3**	30
Figure S 28. IR spectrum of compound **4**	31
Figure S 29. HR-ESI-MS spectra of compound **4**	32
Figure S 30. $^{1H-NMR}$ (600 MHz, DMSO-d6) of compound **4**	33
Figure S 31. $^{13C-NMR}$ (150 MHz, DMSO-d6) of compound **4**	34
Figure S 32. DEPT of compound **4**	35
Figure S 33. HSQC of compound **4**	36
Figure S 34. $^{1H-1H}$ COSY of compound **4**	37
Figure S 35. HMBC of compound **4**	38
Figure S 36. NOESY of compound **4**	39

Table S1. ^{1}H nuclear magnetic resonance (NMR) data (600 MHz, DMSO-d_6) of compounds **5-11**.

Position	**5**	**6**	**7**	**8**	**9**	**10**	**11**
2				6.16 s			
4					5.57 dd (5.5, 3.7)		
5	3.41 dd (14.7, 8.1)	3.45 s	3.18 s	3.22 d (14.0)	3.05 dd (14.9, 3.5) & 2.66 dd (14.9, 5.7)	3.15 d (7.1)	3.37 d (7.5)
2′, 6′	7.52 d (8.6)	7.53 d (8.8)	7.51 d (8.8)	7.51 d (8.8)	7.49 d (8.6)	7.43 d (8.6)	7.51 d (8.8)
3′, 5′	6.59 d (8.6)	6.89 d (8.8)	6.90 (8.9)	6.90 (8.9)	6.81 (8.8)	6.82 (8.8)	6.89 (9.0)
2′′	6.88 d (8.8)	6.57 s	6.42 d (8.9)	6.37 br.s	6.48 d (1.8)	6.86 d (1.7)	6.38 d (2.0)
3′′	6.51 d (8.4)						
5′′	6.51 d (8.4)	6.51 d (8.1)	6.48 d (8.4)	6.52 d (8.2)	6.53 d (8.1)	6.69 d (8.3)	6.54 d (8.3)
6′′	6.88 d (8.8)	6.50 dd (8.1, 4.4)	6.54 dd (18.4, 1.8)	6.52 d (8.3)	6.57 dd (8.1, 2.0)	6.79 dd (8.3, 1.7)	6.49 dd (8.3, 2.2)
1′′′		2.80 m	2.65 dd (16.7, 5.5) & 2.42 dd (16.7, 5.5)	3.00 d (6.6)	3.02 m	3.15 m	3.00 m
2′′′		4.45 t (9.4)	3.56 t (5.7)	5.00 m	5.06 t (8.6)	5.21 t (6.1)	5.01 t (6.1)
4′′′		1.07 s	1.23 s	1.62 s	1.58 s	1.65 s	1.63 s
5′′′		1.07 s	1.07 s	1.53 s	1.53 s	1.61 s	1.54 s
COOCH3	3.74 s	3.74 s	3.75 s				
2/4-OH	10.59 br. s [2-OH]	10.57 br. s [2-OH]	10.03 br. s [2-OH]	7.89 br. s [4-OH]		10.18 br. s [2-OH]	10.52 br. s [2-OH]
4′-OH	9.97 br. s	9.98 br.s		10.22 br,s	9.82 br. s	9.21 br. s	9.97 br. s
4′′-OH	9.25 br. s			9.11 br.s	9.05 br. s	8.90 br. s	9.17 br. s [2-OH]
3′′′-OH		4.54 br. s					

* Sample dissolved in DMSO-d_6.

Table S2. ^{13}C-NMR Data of compounds **5-11** (DMSO-d_6, 125 MHz).

Position	**5**	**6**	**7**	**8**	**9**	**10**	**11**
1	168.4	168.5	168.5	170.3	169.6	168.0	168.4
2	138.5	136.6	138.7	112.9	136.8	131.7	138.6
3	127.9	127.9	127.9	164.1	128.3	127.4	127.8
4	85.1	70.4	85.2	108.2	78.3	102.0	85.2
5	38.4	38.7	38.4	43.7	38.6	40.4	38.5
1'	121.4	121.5	121.5	121.3	122.3	120.6	121.6
2′, 6′	129.2	129.3	129.4	131.1	129.4	129.4	129.2
3′, 5′	116.3	116.3	116.3	116.2	116.0	116.1	116.2
4′	158.3	158.3	158.3	160.5	158.2	160.0	158.3
1′′	123.6	124.9	120.1	124.8	125.6	125.3	123.6
2′′	131.6	129.9	132.0	130.8	131.0	130.7	131.4
3′′	115.1	127.3	124.8	126.8	127.1	126.3	127.0
4′′	156.7	159.2	152.1	154.0	153.9	153.8	154.2
5′′	115.1	115.7	116.2	114.6	114.7	115.3	114.5
6′′	131.6	127.1	129.2	128.6	130.0	128.0	128.9
1′′′		30.2	31.4	28.0	28.3	29.2	28.0
2′′′		89.5	68.4	123.4	123.2	123.1	122.8
3′′′		108.3	77.4	131.7	131.5	130.7	131.8
4′′′		25.3	26.1	25.9	26.0	25.9	26.0
5′′′		26.4	20.4	17.9	18.1	18.0	18.0
<u>C</u>OOCH$_3$/COOH	170.2	170.2	170.2			172.1	170.3
COO**<u>CH$_3$</u>**	53.9	54.0	53.9				53.9

* Sample dissolved in DMSO-d_6.

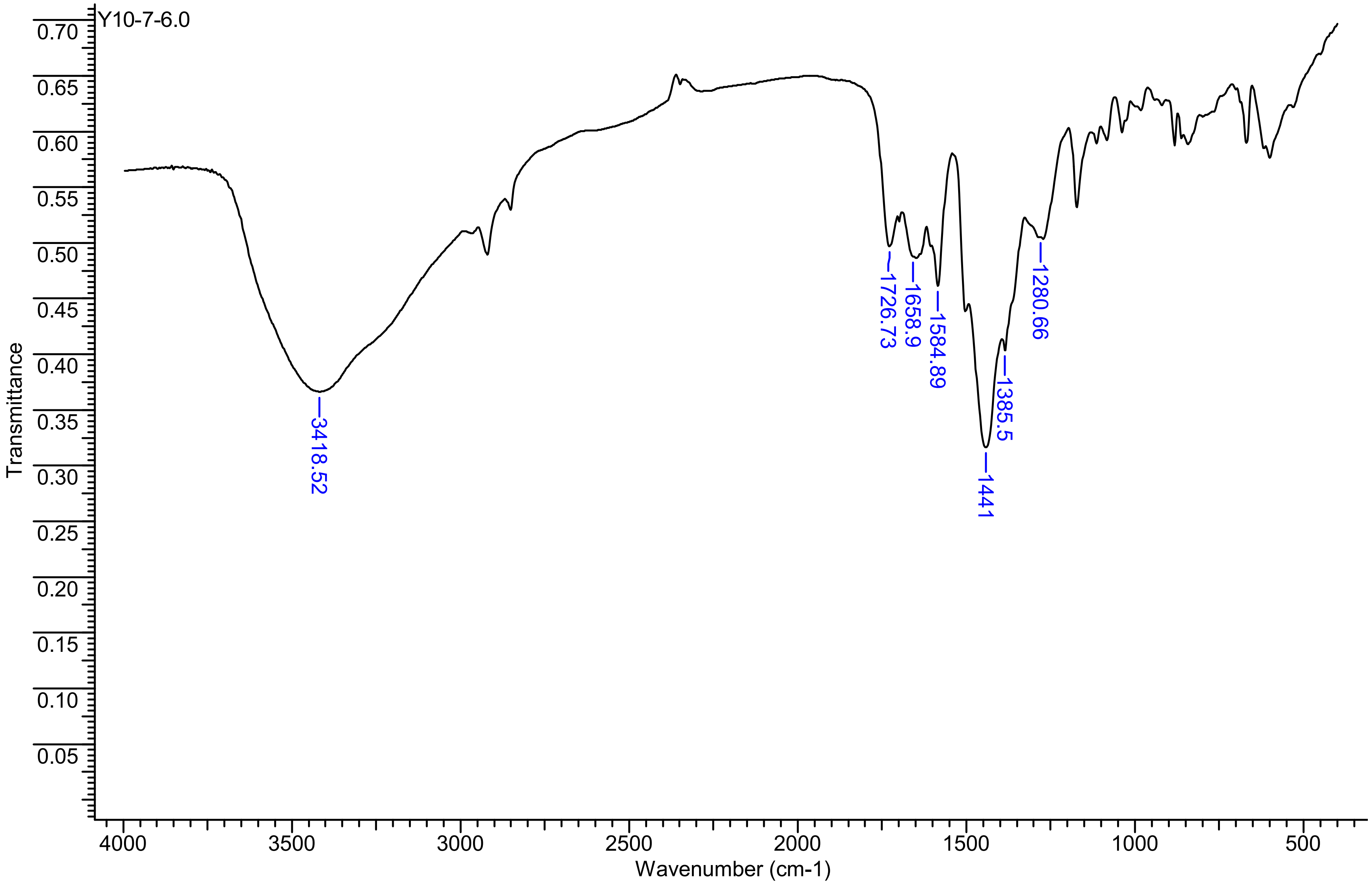

Figure S 1. Infrared (IR) spectrum of compound **1.**

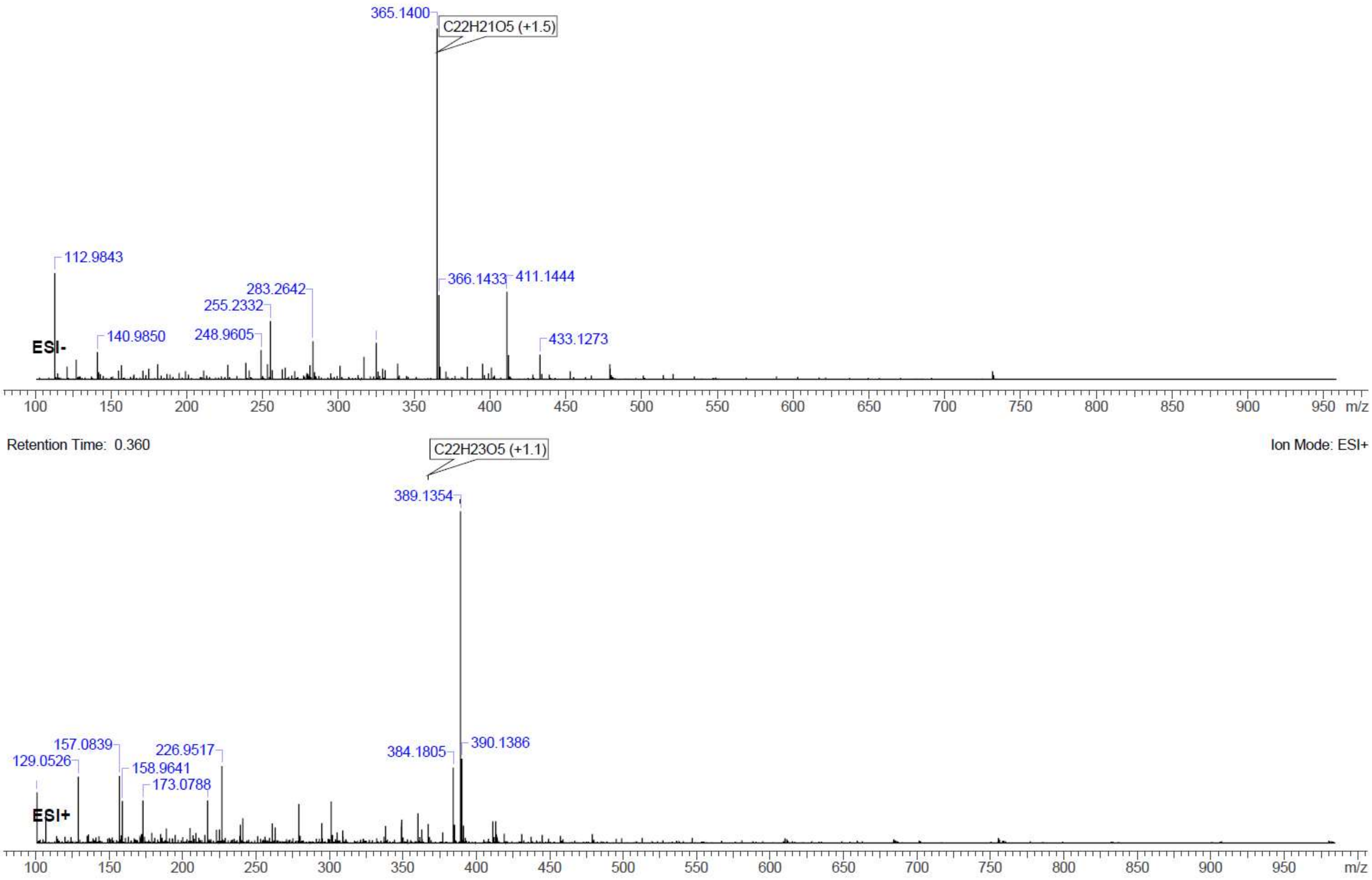

Figure S 2. High-resolution electrospray ionisation mass spectrometry (HR-ESI-MS) spectra of compound **1.**

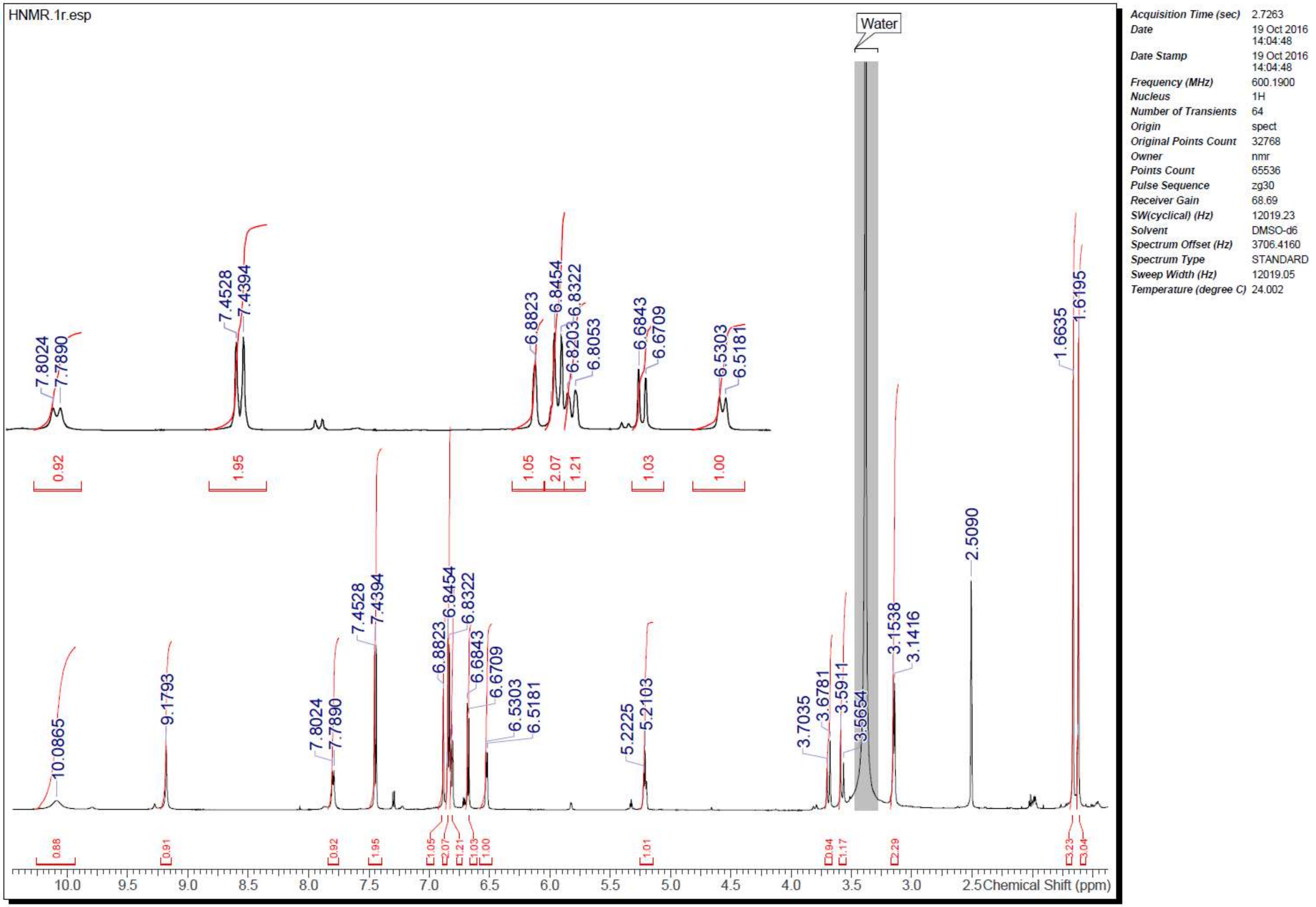

Figure S 3. ^{1}H-NMR (600 MHz, DMSO-d_6) of compound **1.**

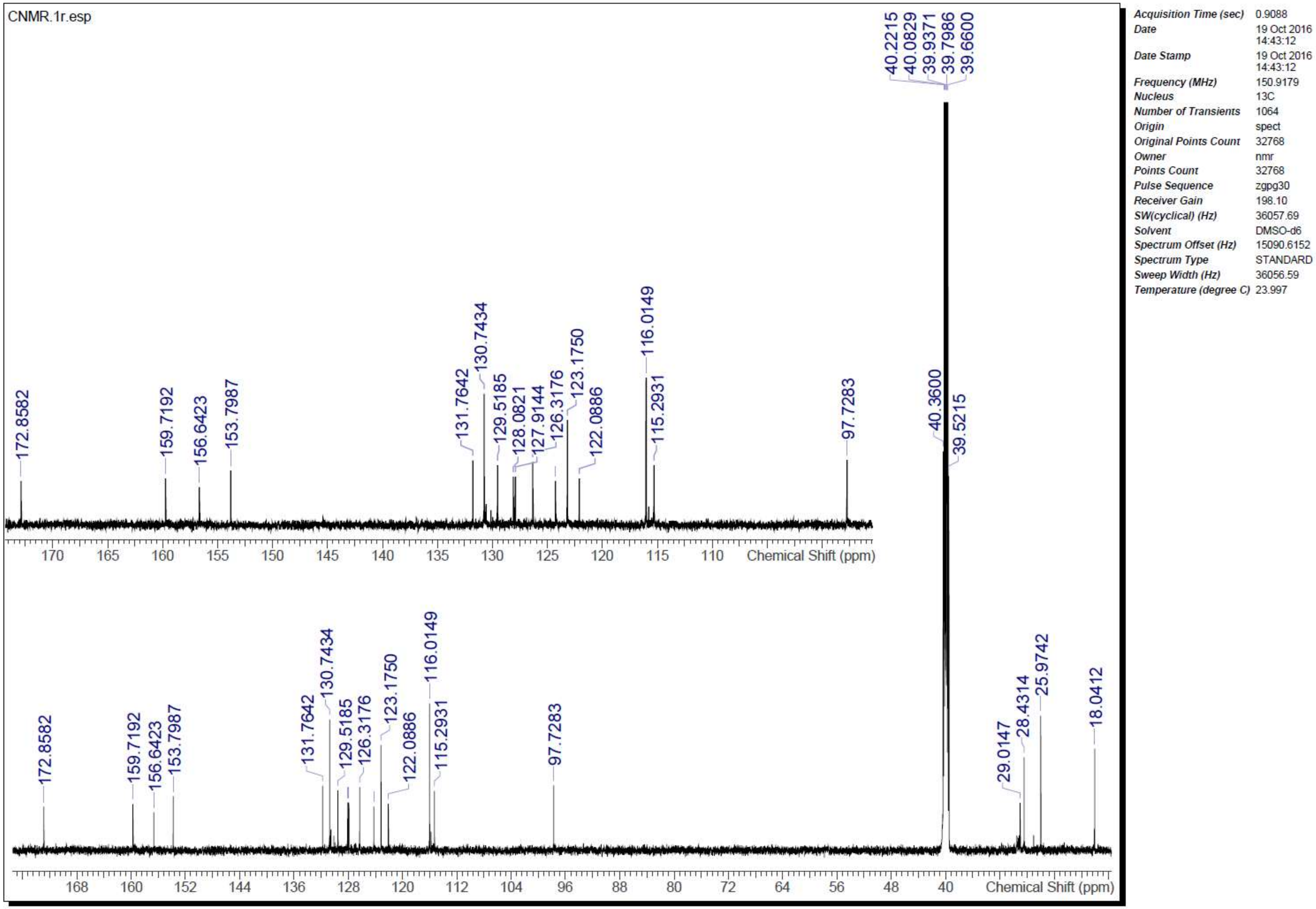

Figure S 4. ^{13}C-NMR (150 MHz, DMSO-d_6) of compound **1.**

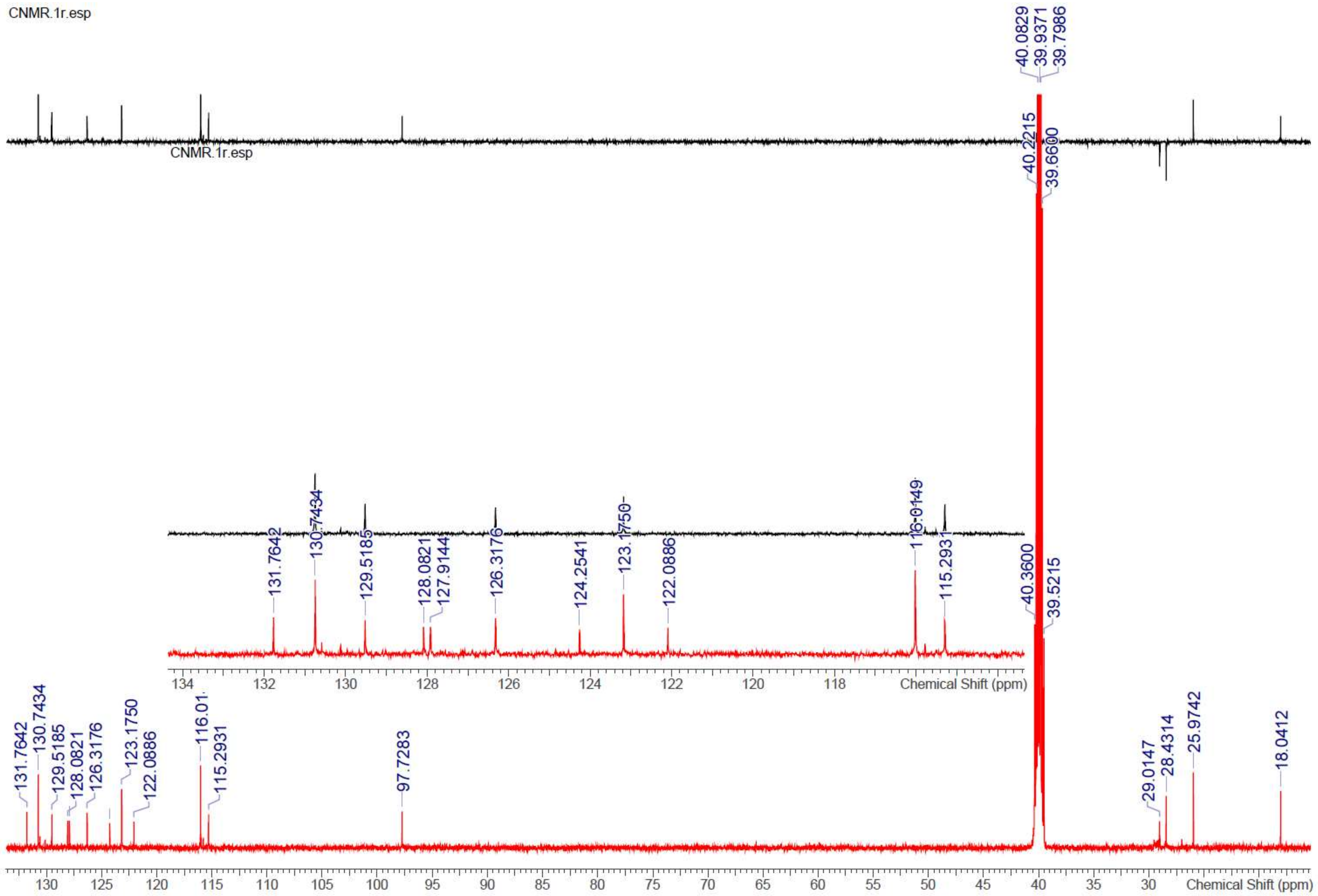

Figure S 5. DEPT of compound **1.**

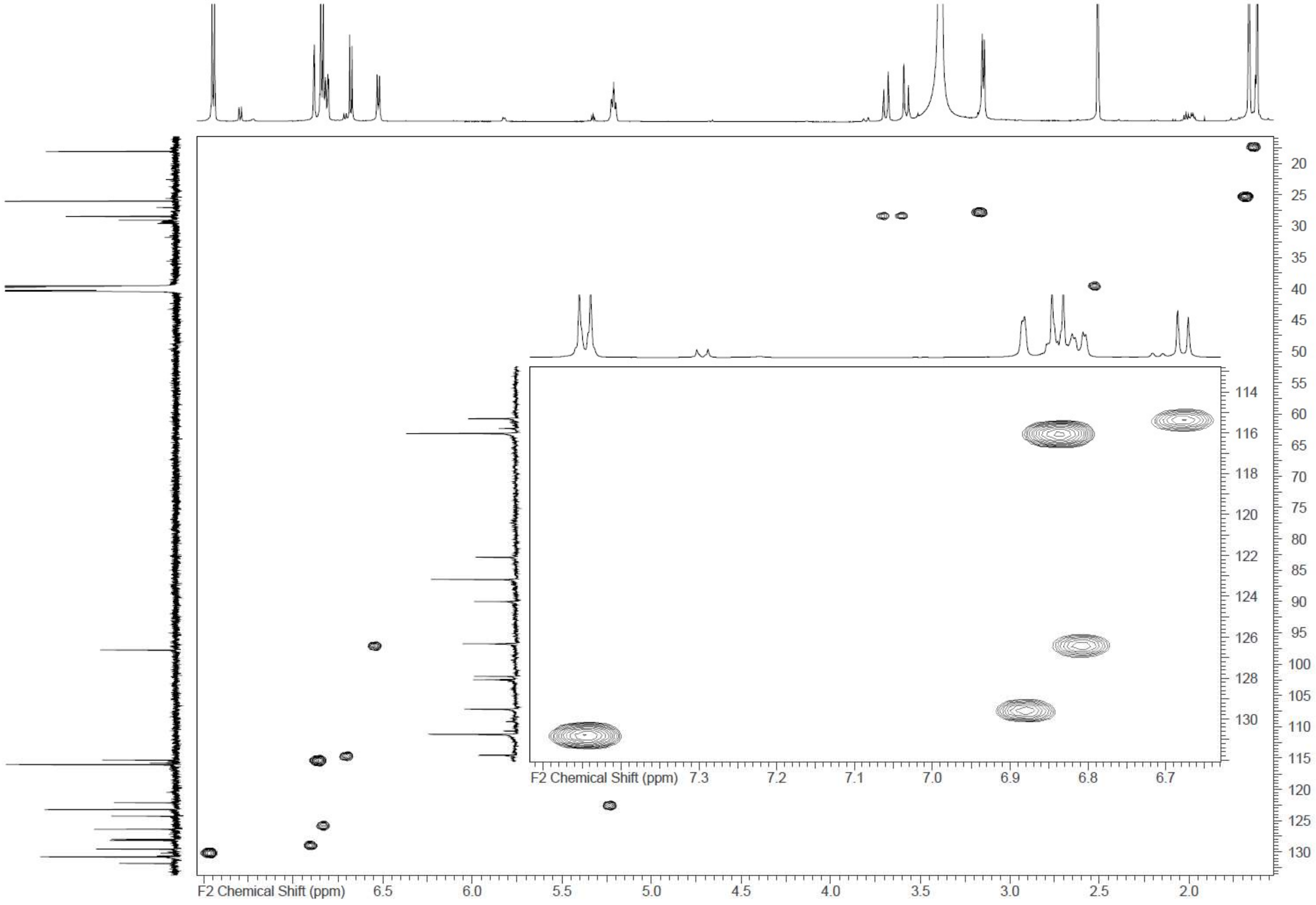

Figure S 6. Heteronuclear single quantum coherence spectroscopy (HSQC) of compound **1.**

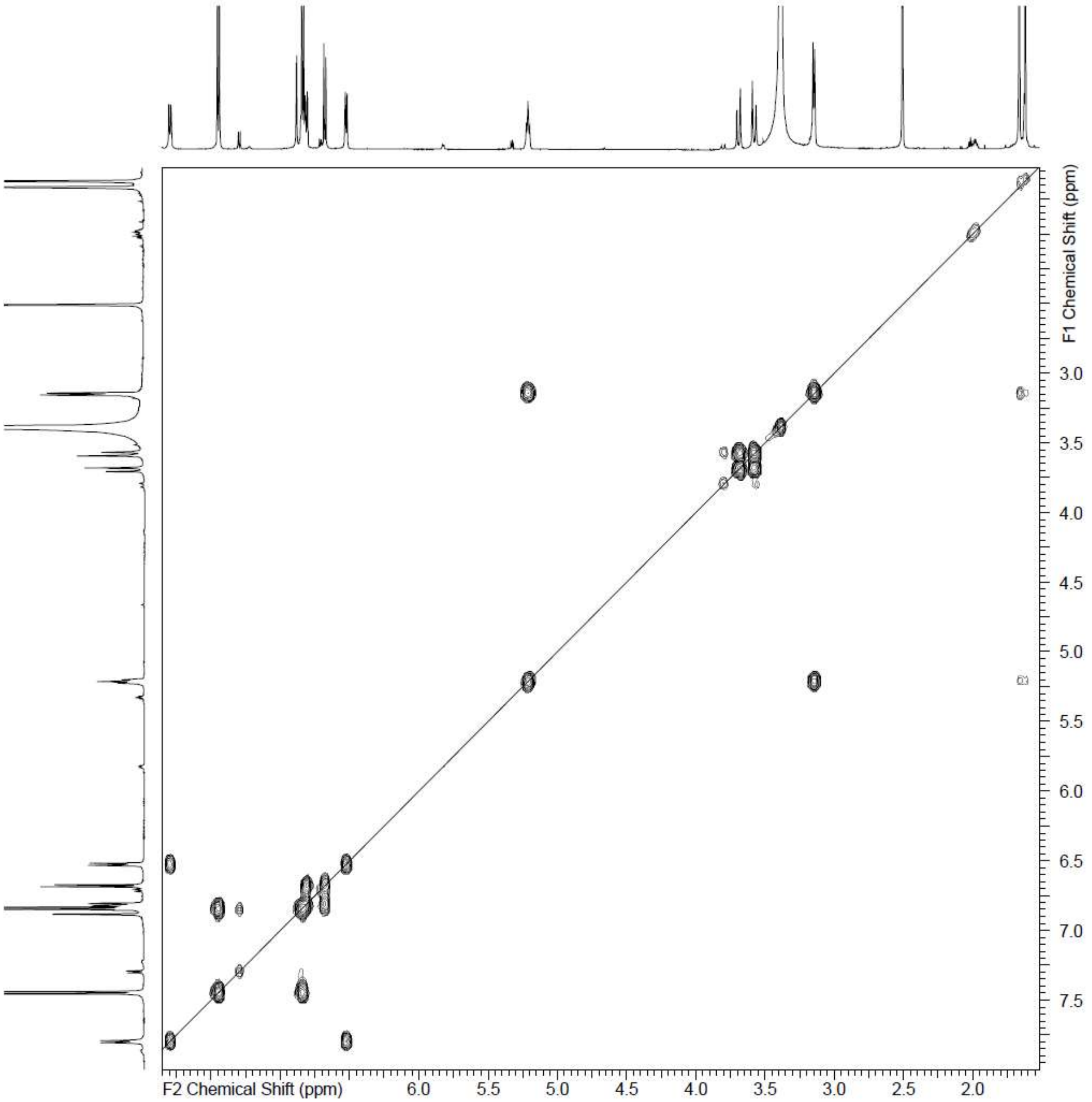

Acquisition Time (sec)	(0.0852, 0.0214)
Comment	5 mm PABBO BB/19F-1H/D Z-GRD Z114607/0133
Date	18 Nov 2016 20:05:52
File Name	C:\Users\Administrator\Desktop\yanxia-20161121\yanxia-20161121\Y10-7-6-1118\4\pdata\1\2rr
Frequency (MHz)	(600.1936, 600.1936)
Nucleus	(1H, 1H)
Number of Transients	16
Origin	spect
Original Points Count	(1024, 256)
Owner	nmr
Points Count	(2048, 1024)
Pulse Sequence	cosygpmfqf
Solvent	DMSO-d6
Spectrum Type	COSY
Sweep Width (Hz)	(12013.36, 11978.70)
Temperature (degree C)	24.004
Title	

Figure S 7. ^{1}H-^{1}H COSY of compound **1.**

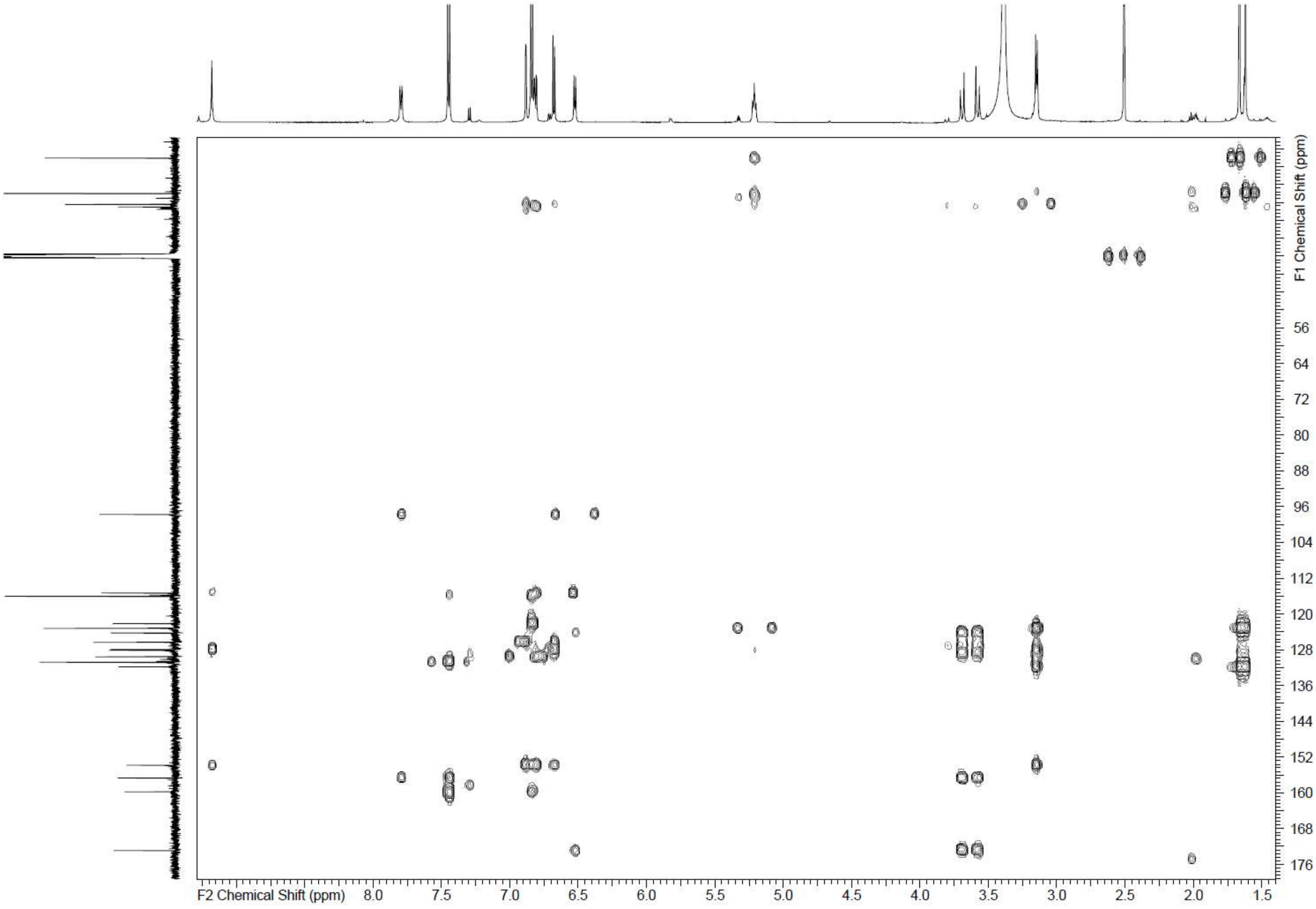

Figure S 8. HMBC of compound **1.**

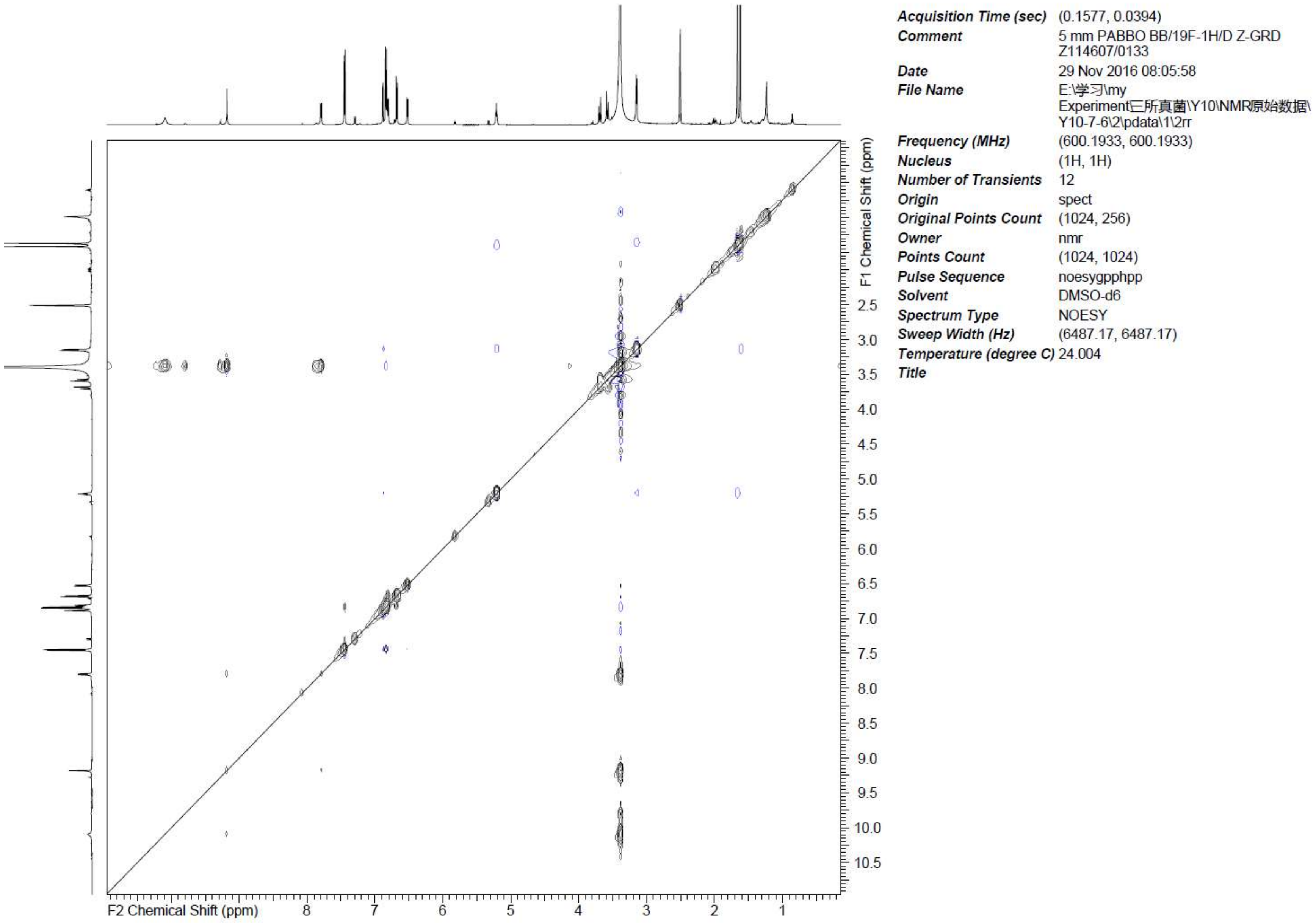

Figure S 9. NOESY of compound **1.**

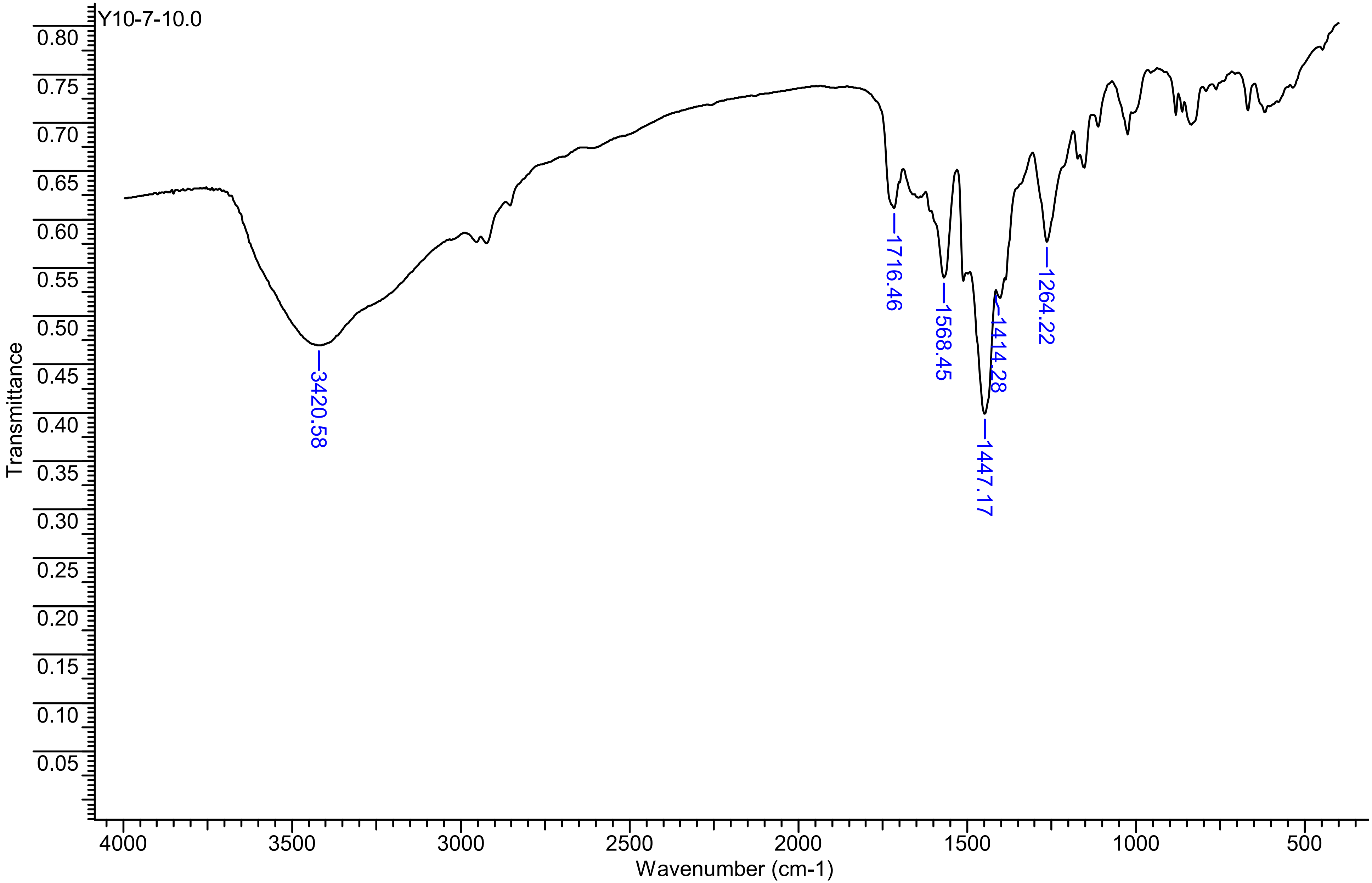

Figure S 10. IR spectrum of compound **2.**

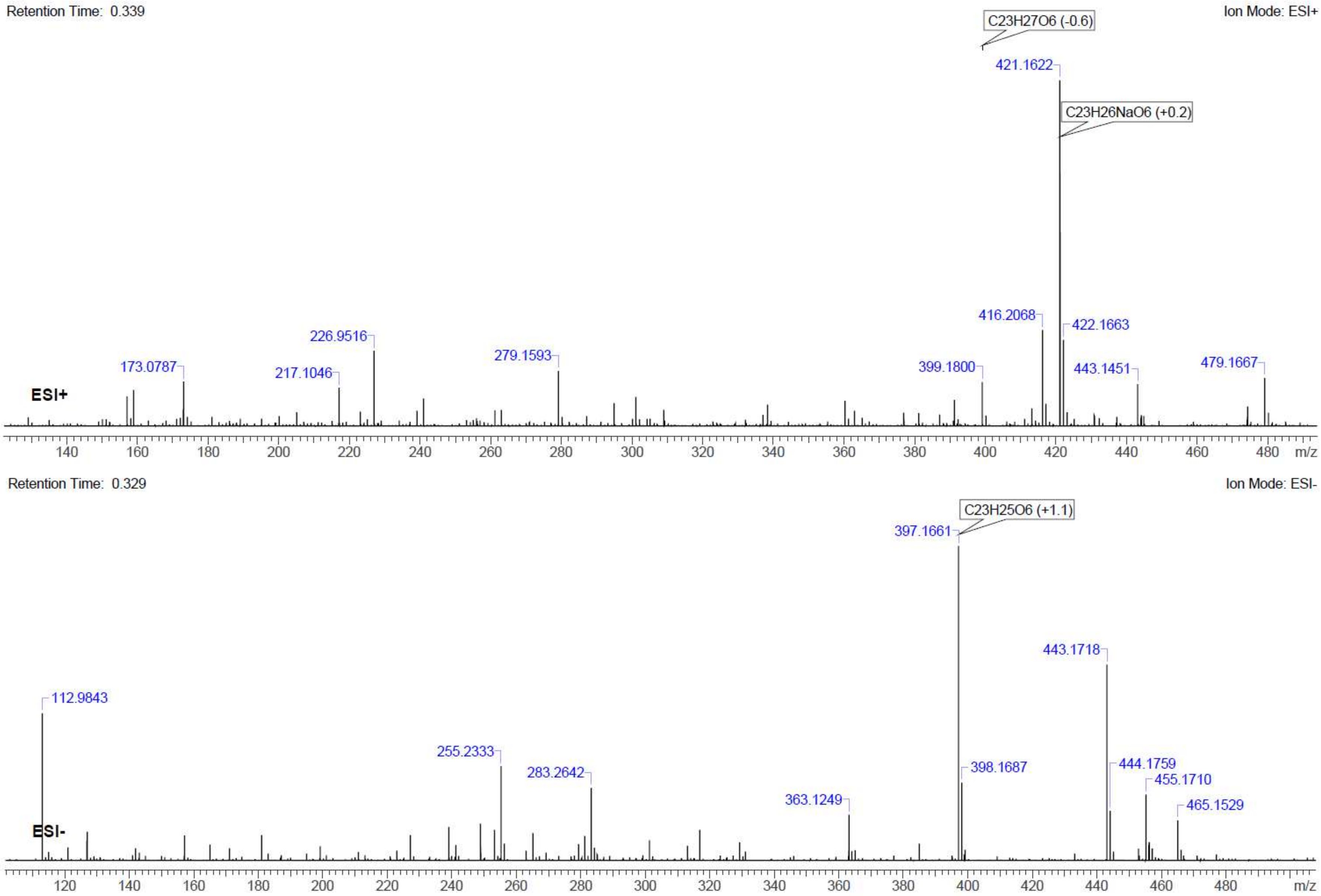

Figure S 11. HR-ESI-MS spectra of compound **2.**

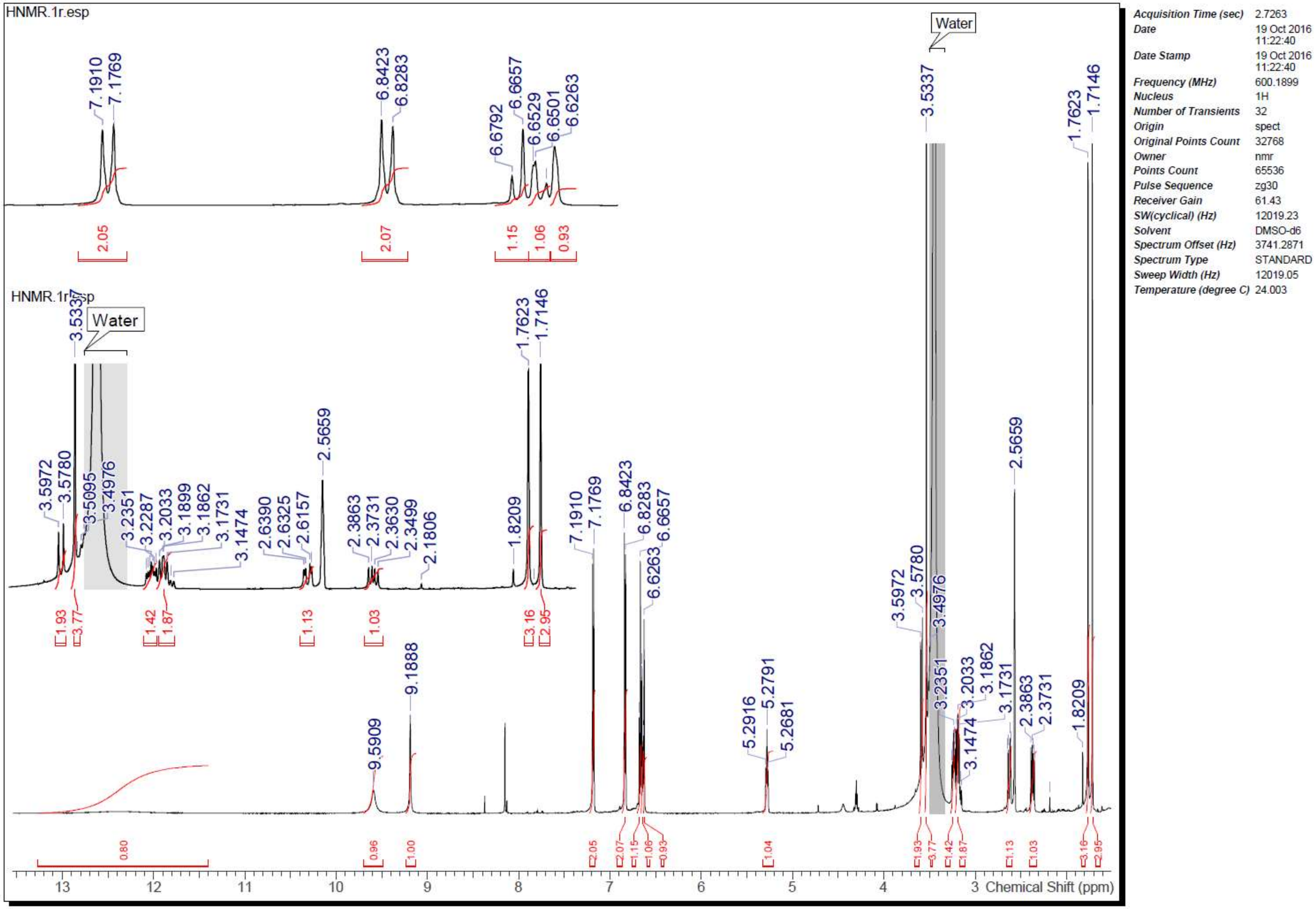

Figure S 12. ^{1}H-NMR (600 MHz, DMSO-d_6) of compound **2.**

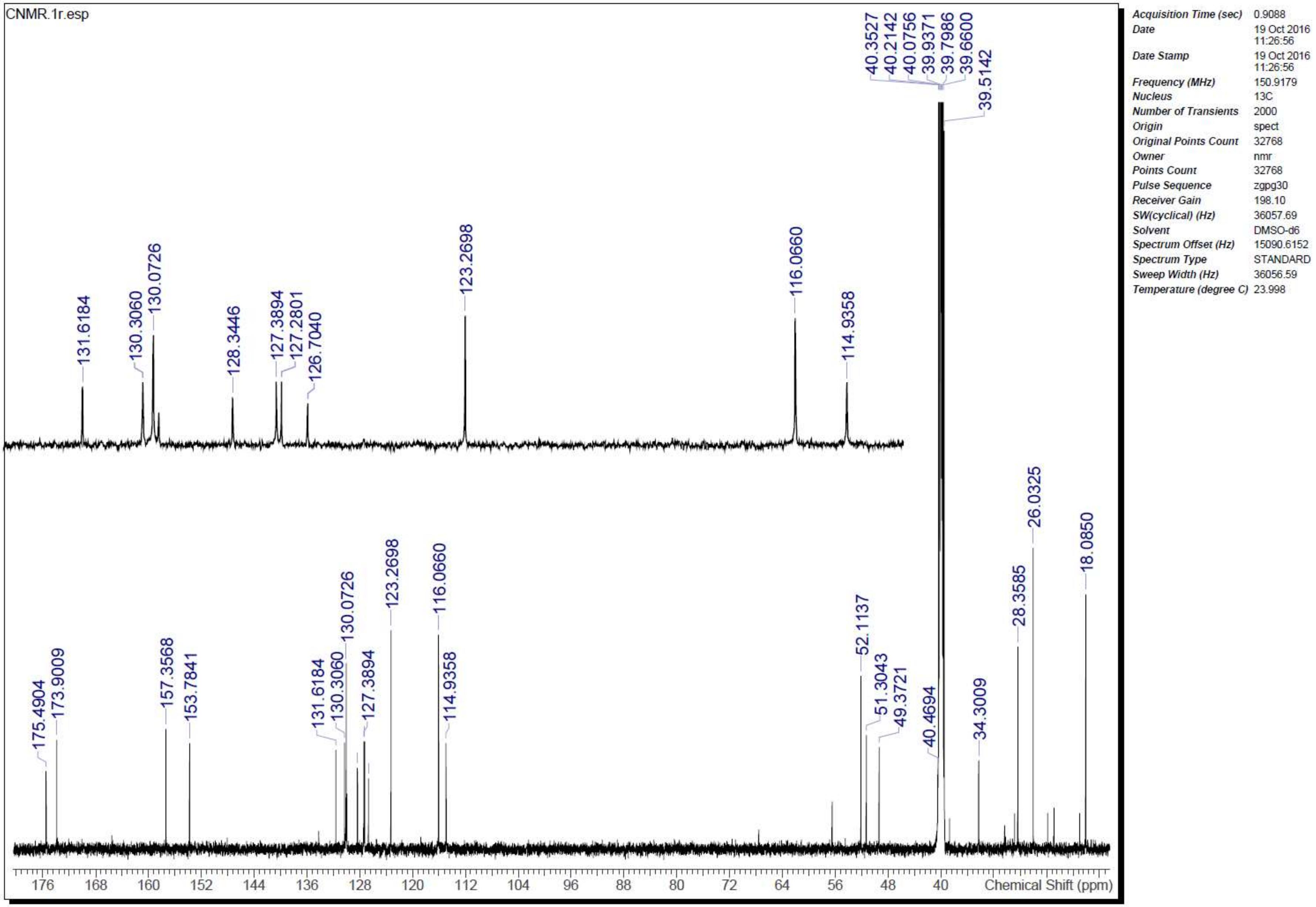

Figure S 13. ^{13}C-NMR (150 MHz, DMSO-d_6) of compound **2.**

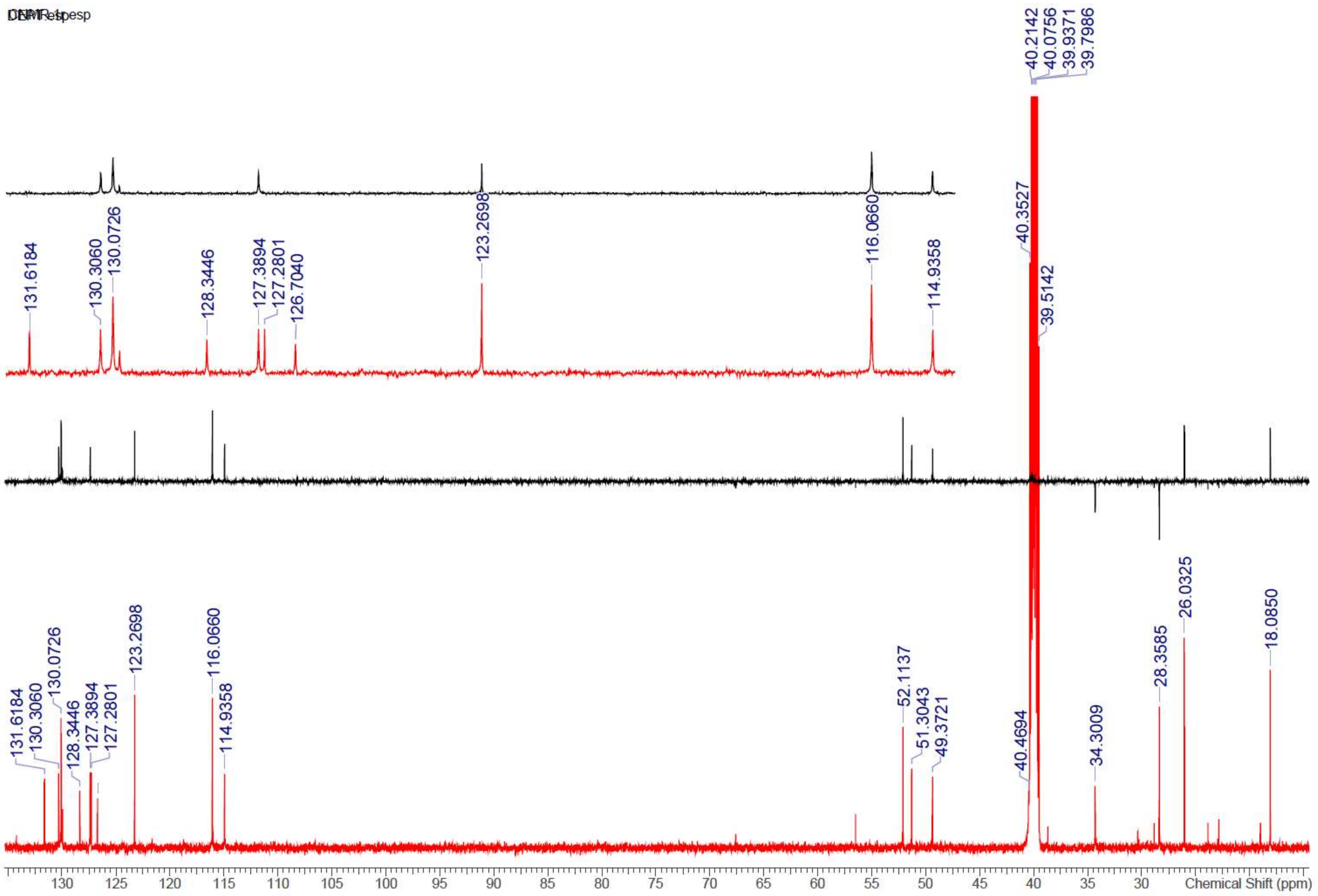

Figure S 14. DEPT of compound **2.**

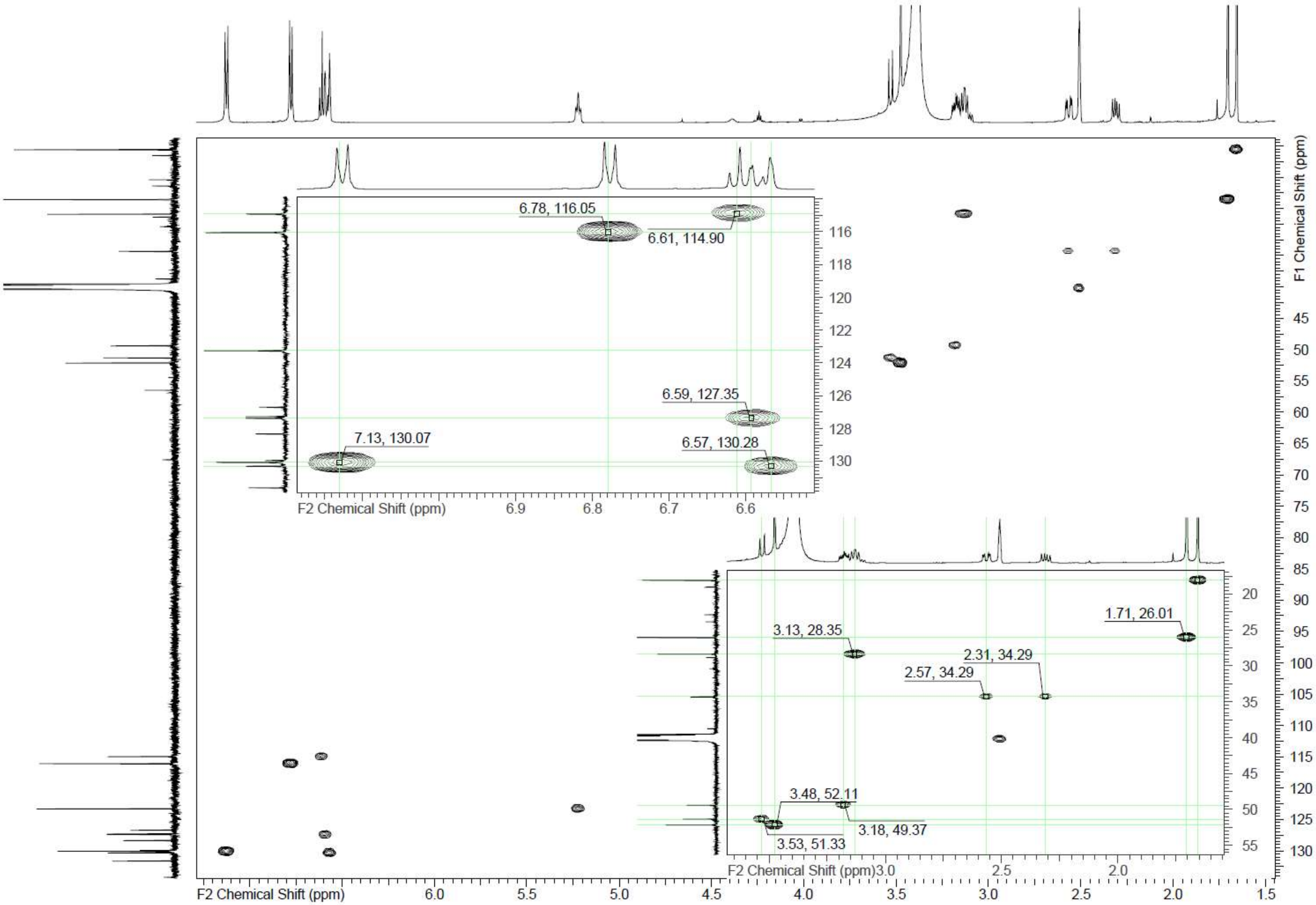

Figure S 15. HSQC of compound **2.**

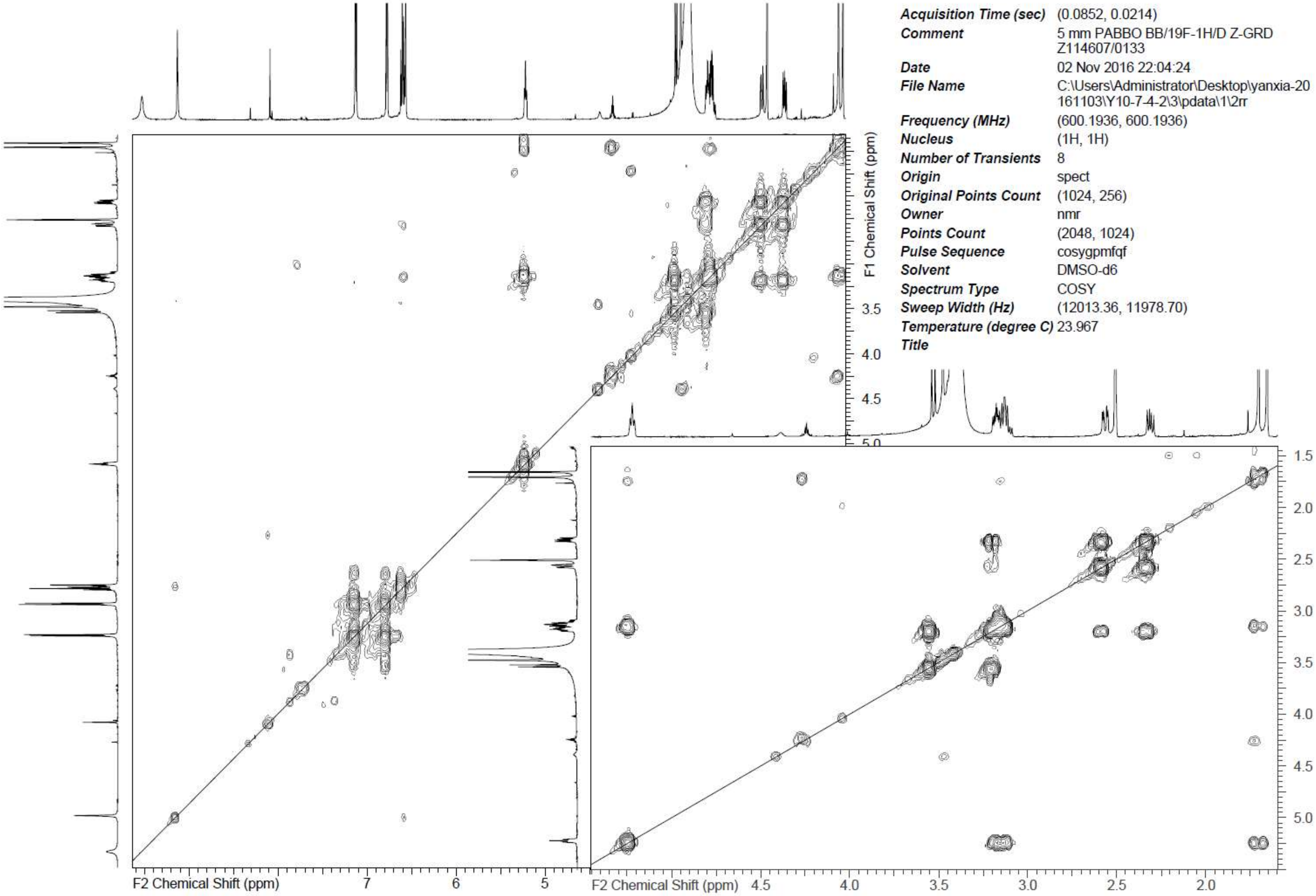

Figure S 16. ^{1}H-^{1}H COSY of compound **2.**

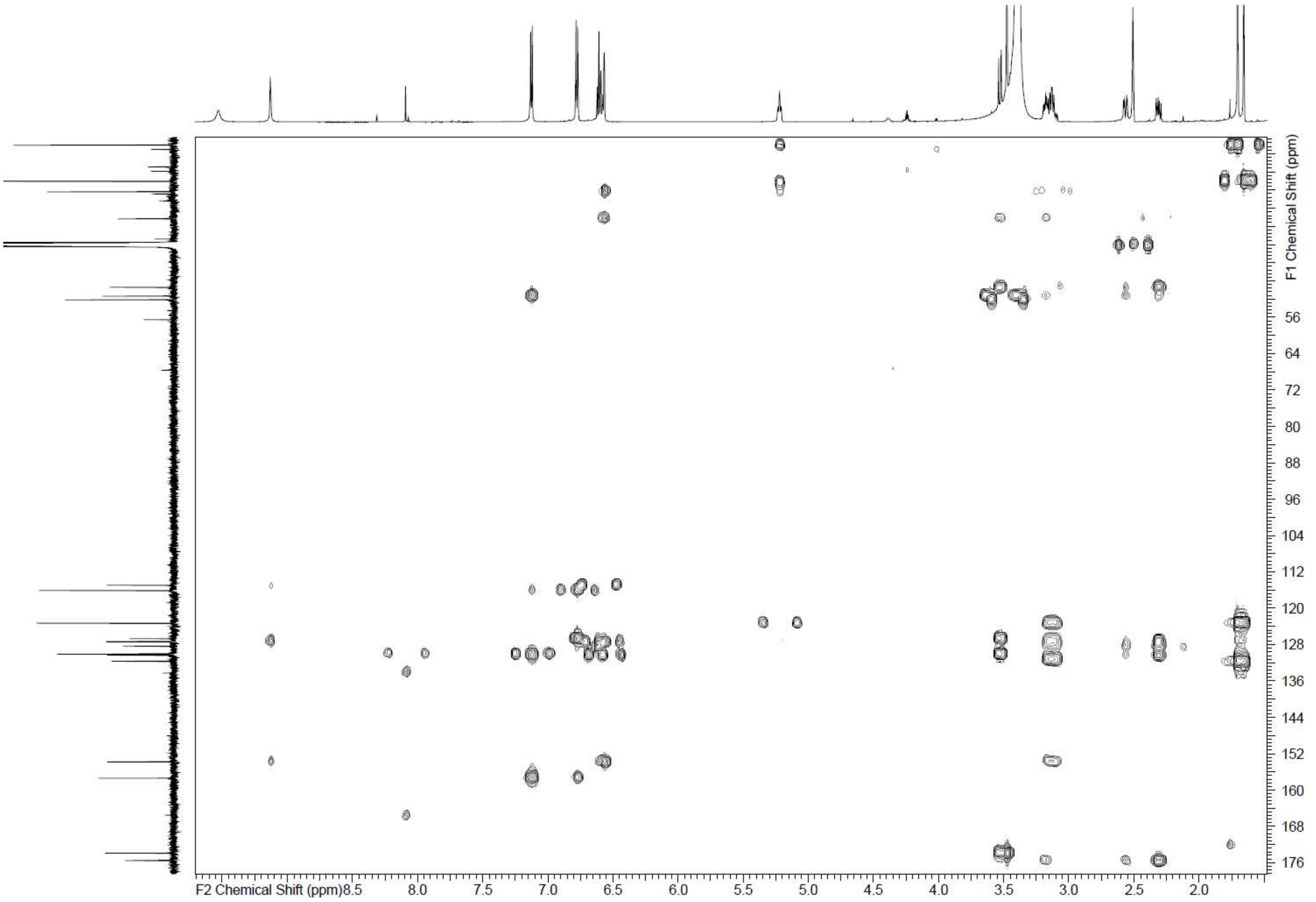

Figure S 17. HMBC of compound **2.**

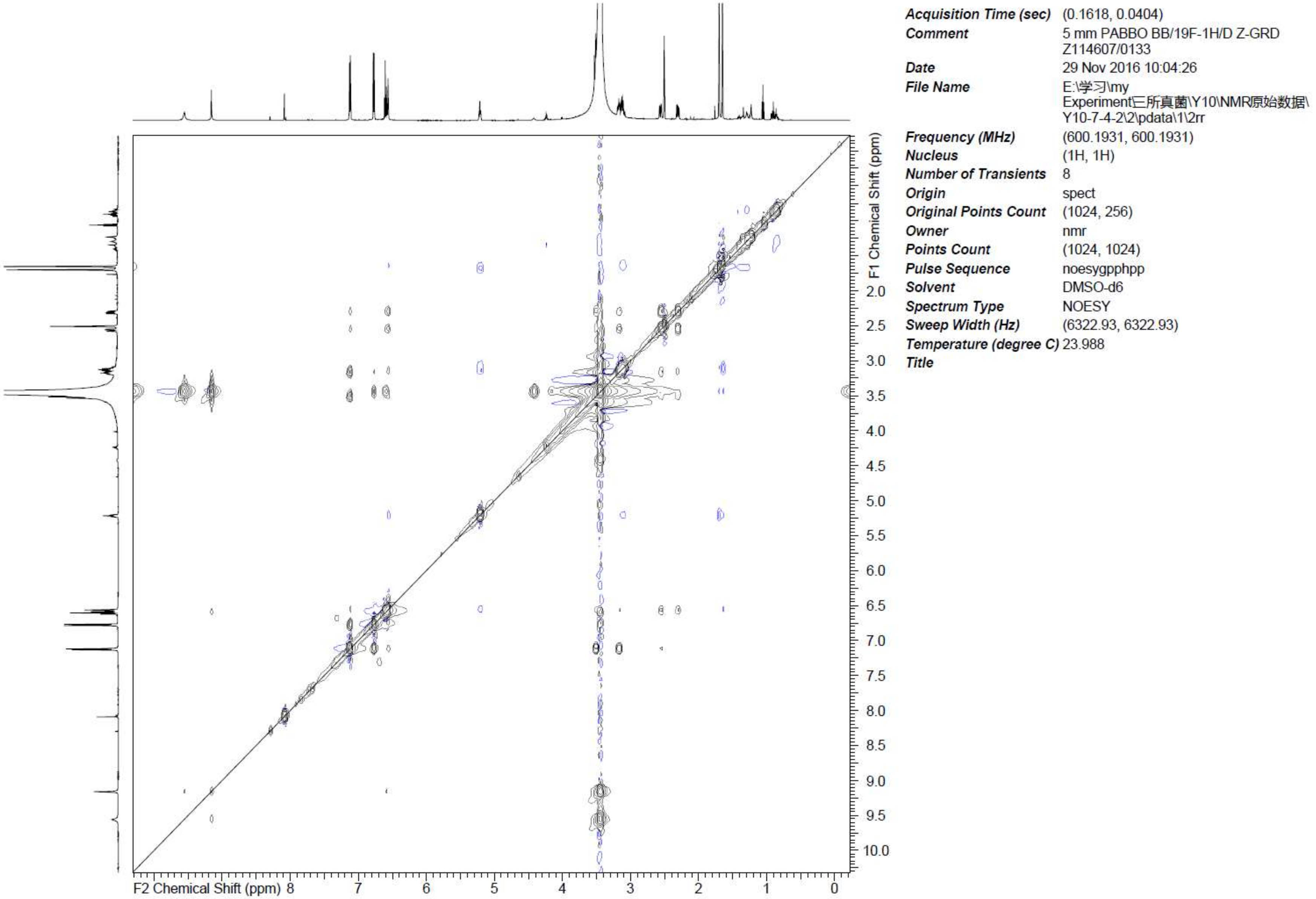

Figure S 18. NOESY of compound **2.**

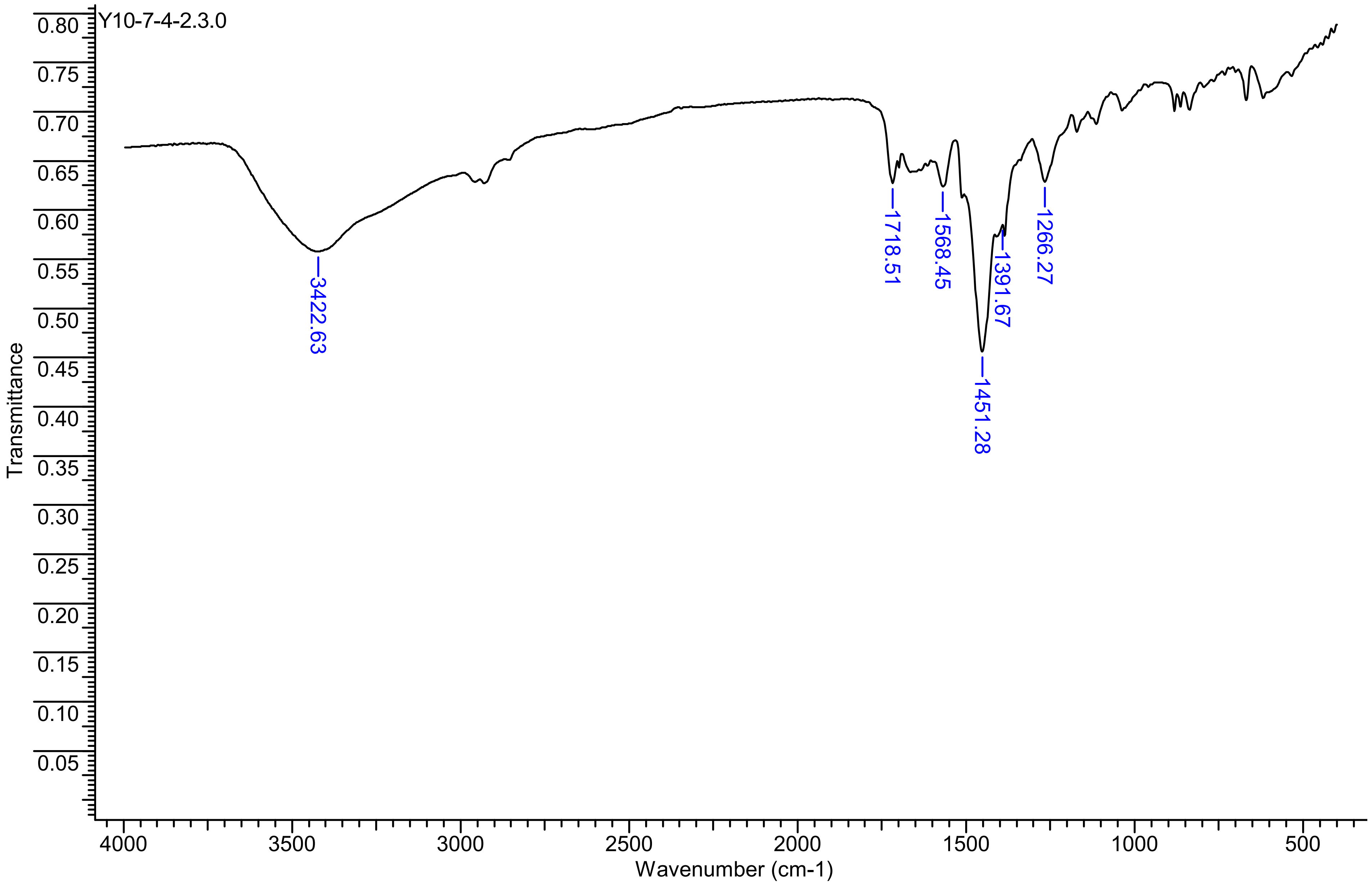

Figure S 19. IR spectrum of compound **3.**

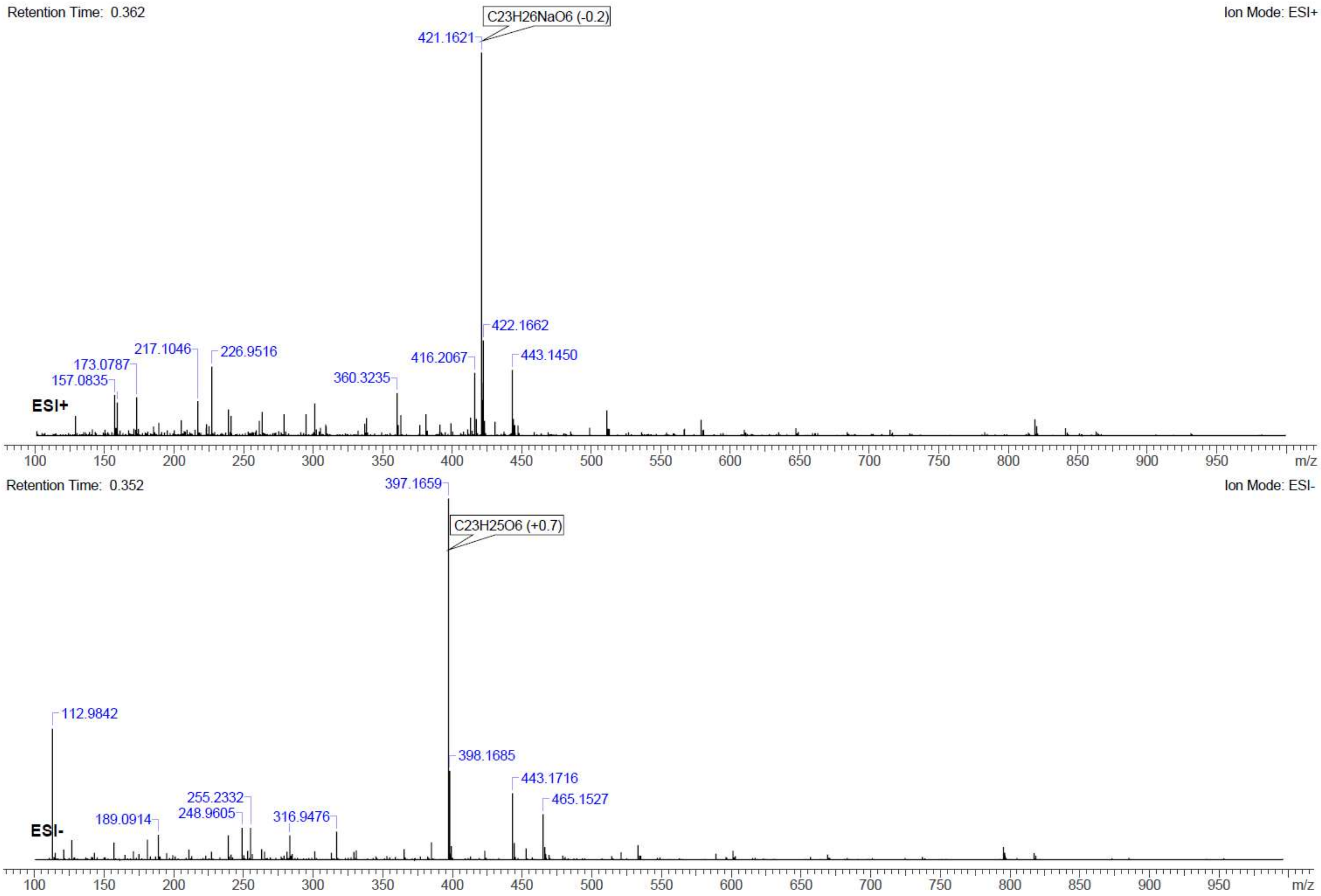

Figure S 20. HR-ESI-MS spectra of compound **3.**

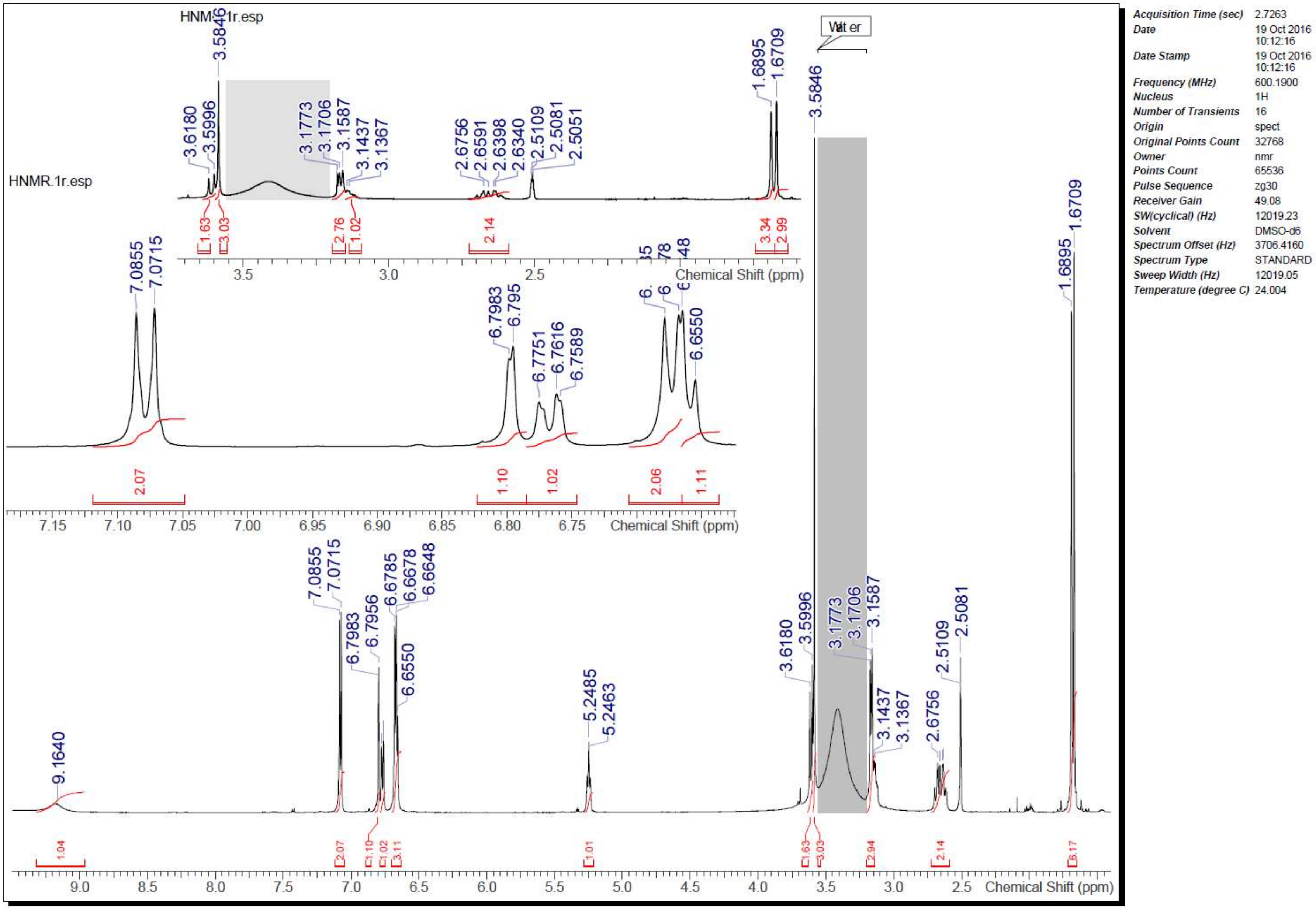

Figure S 21. ^{1}H-NMR (600 MHz, DMSO-d_6) of compound **3.**

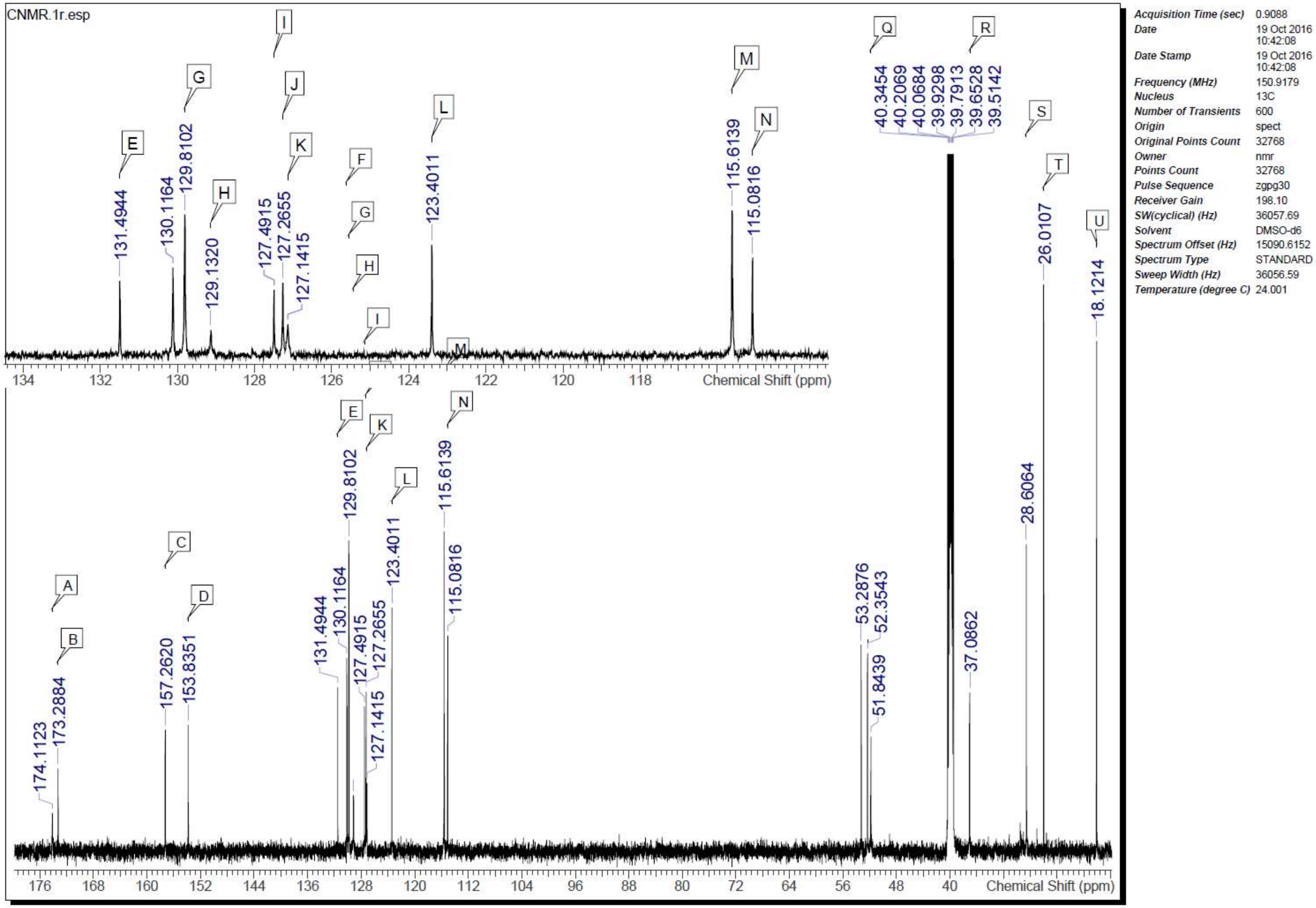

Figure S 22. ^{13}C-NMR (150 MHz, DMSO-d_6) of compound **3.**

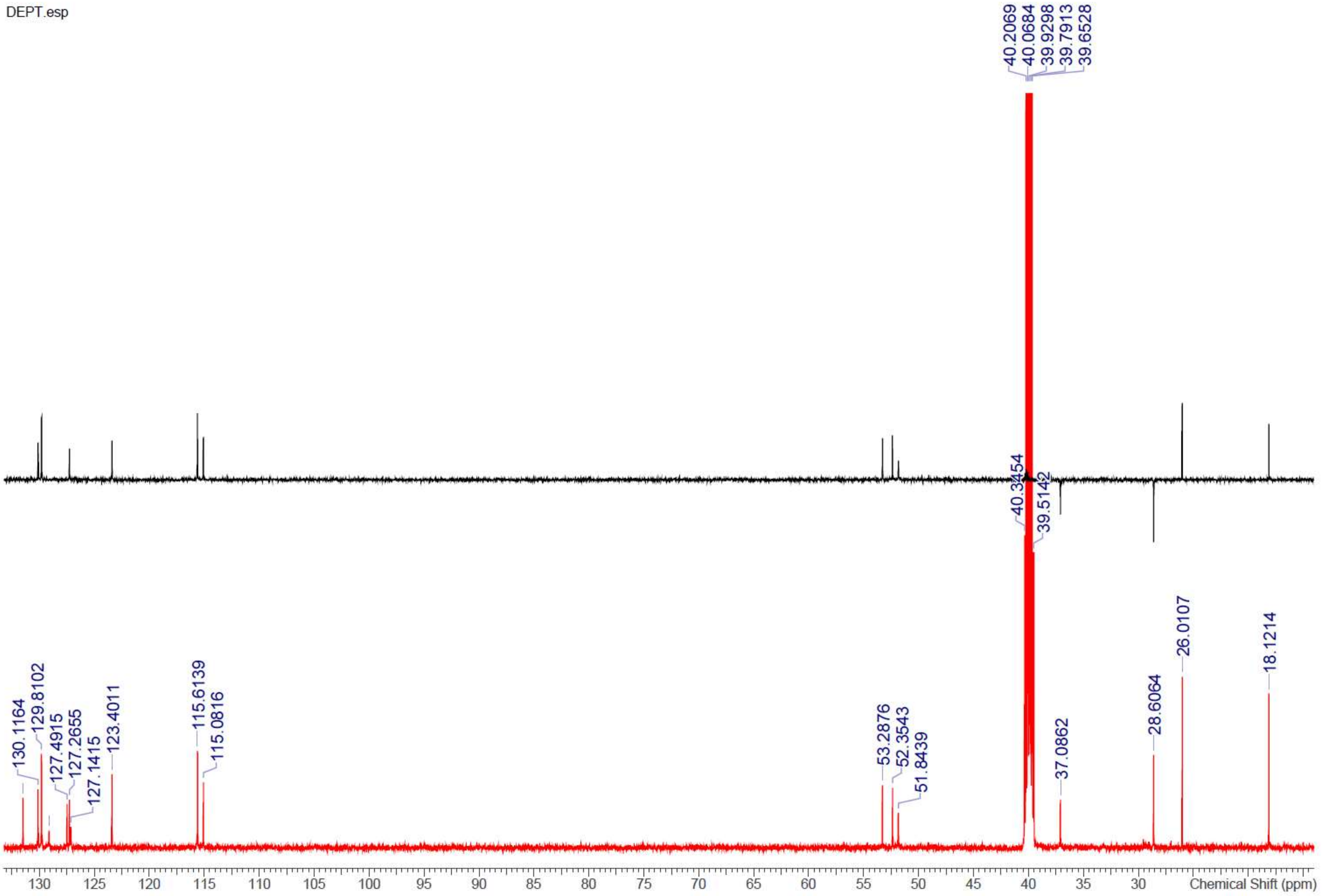

Figure S 23. DEPT of compound **3.**

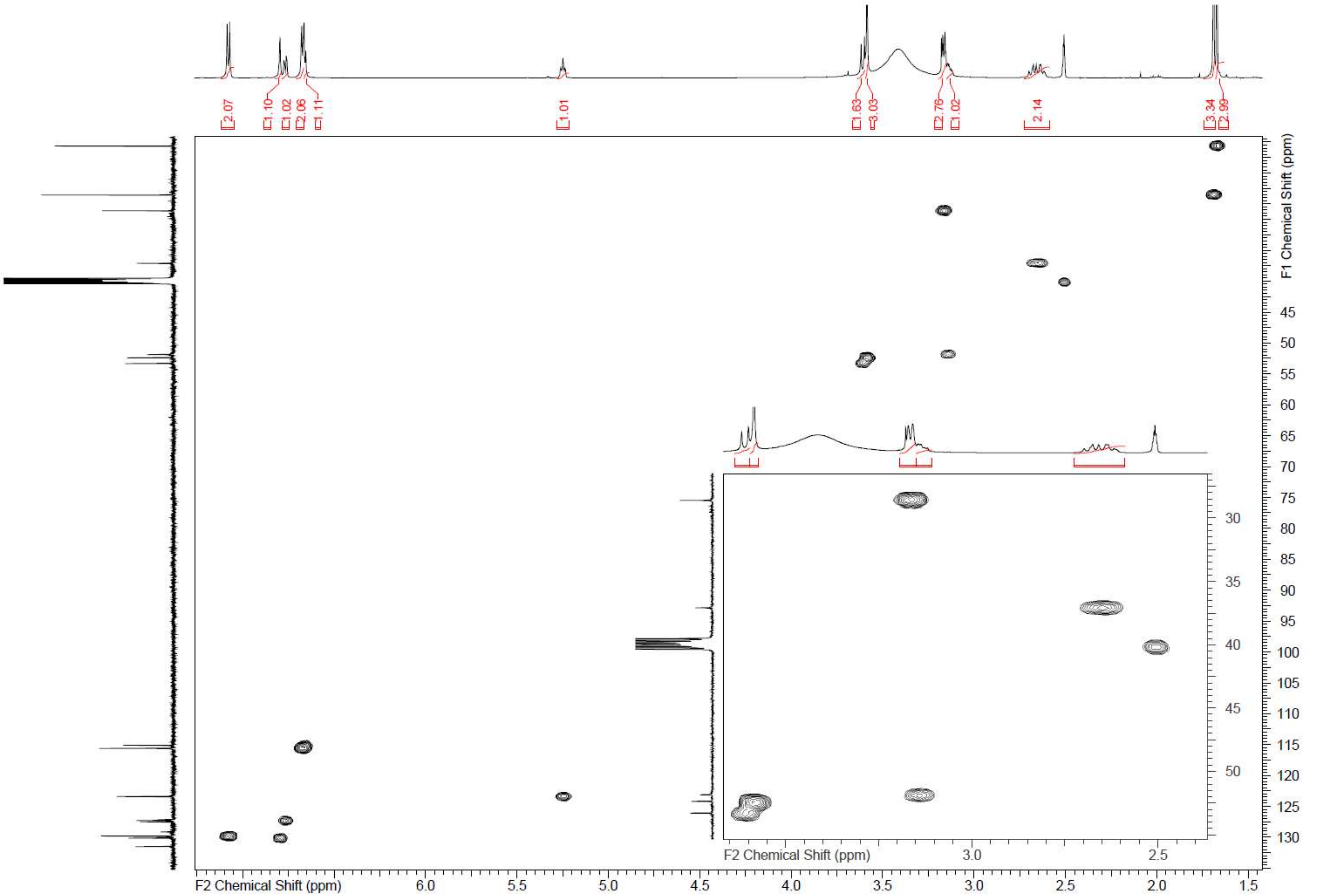

Figure S 24. HSQC of compound **3.**

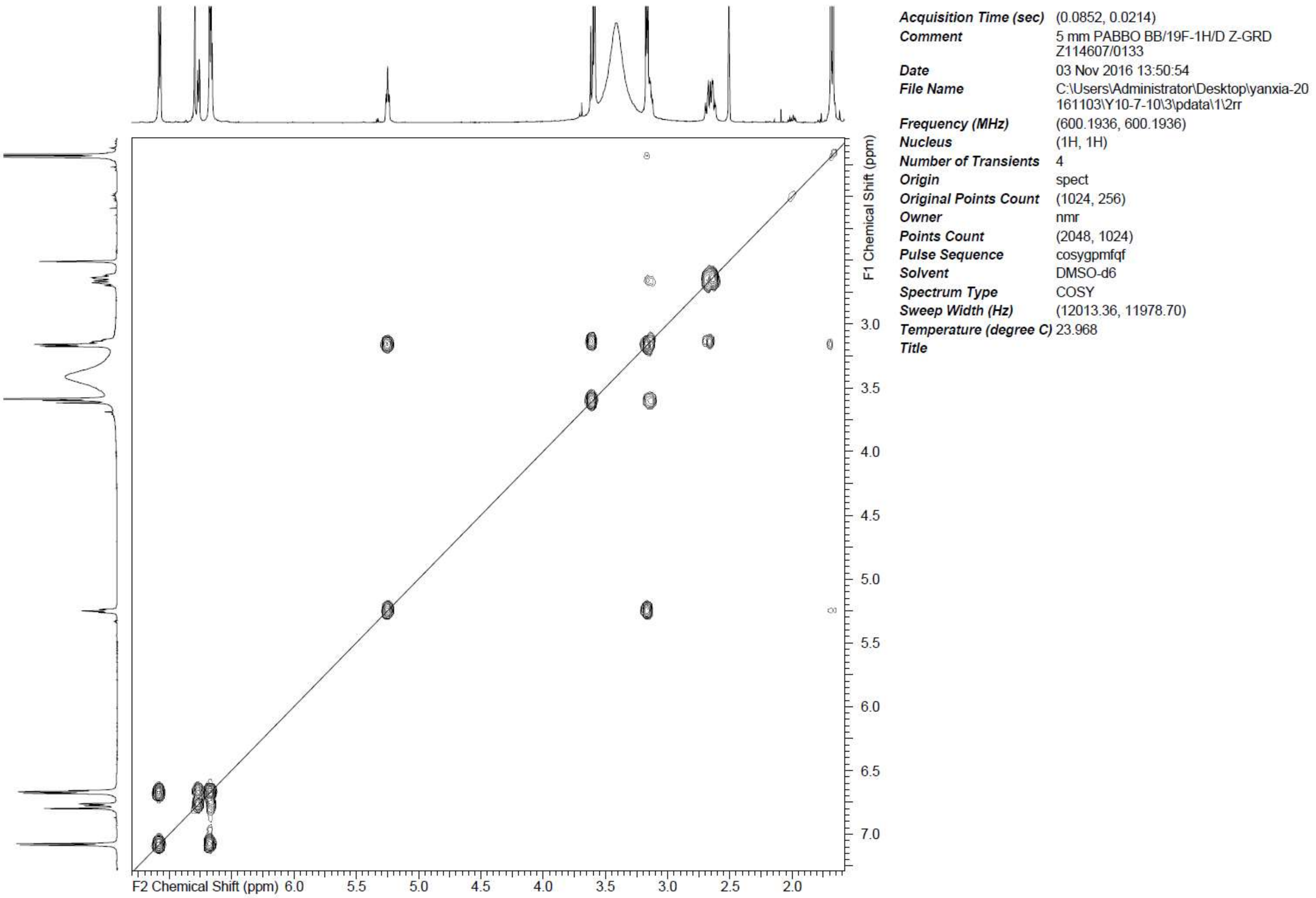

Figure S 25. ^{1}H-^{1}H COSY of compound **3.**

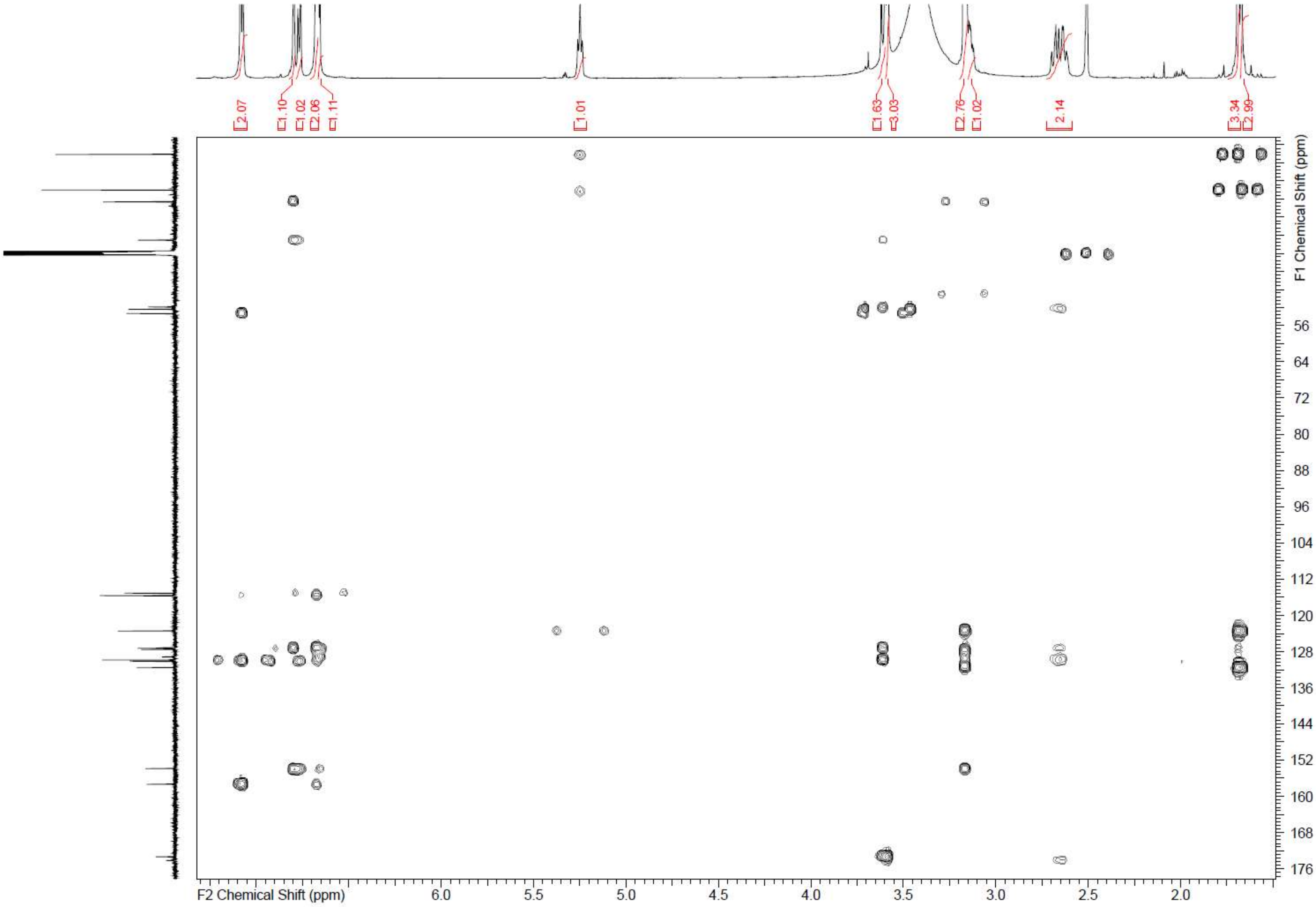

Figure S 26. HMBC of compound **3.**

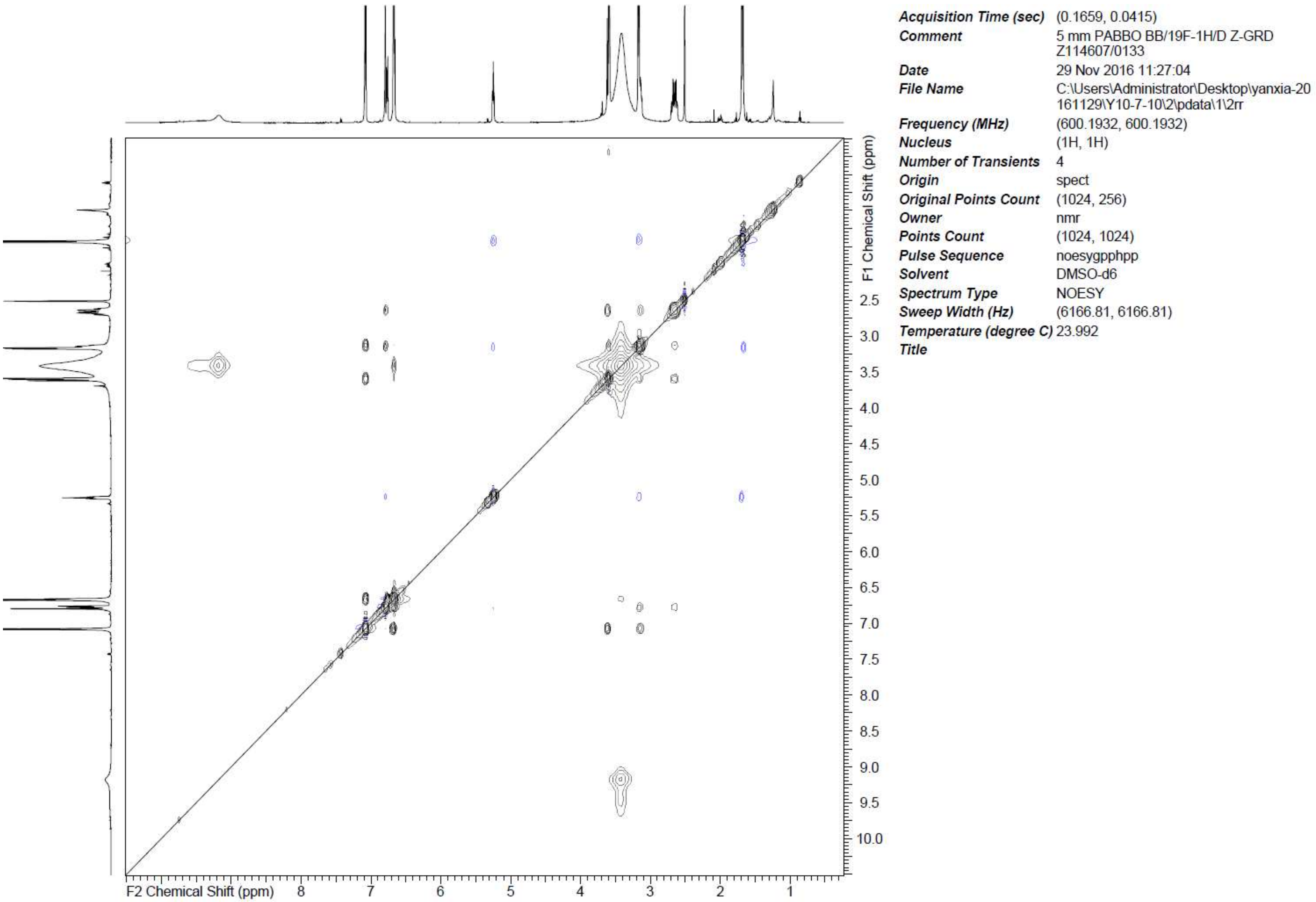

Figure S 27. NOESY of compound **3.**

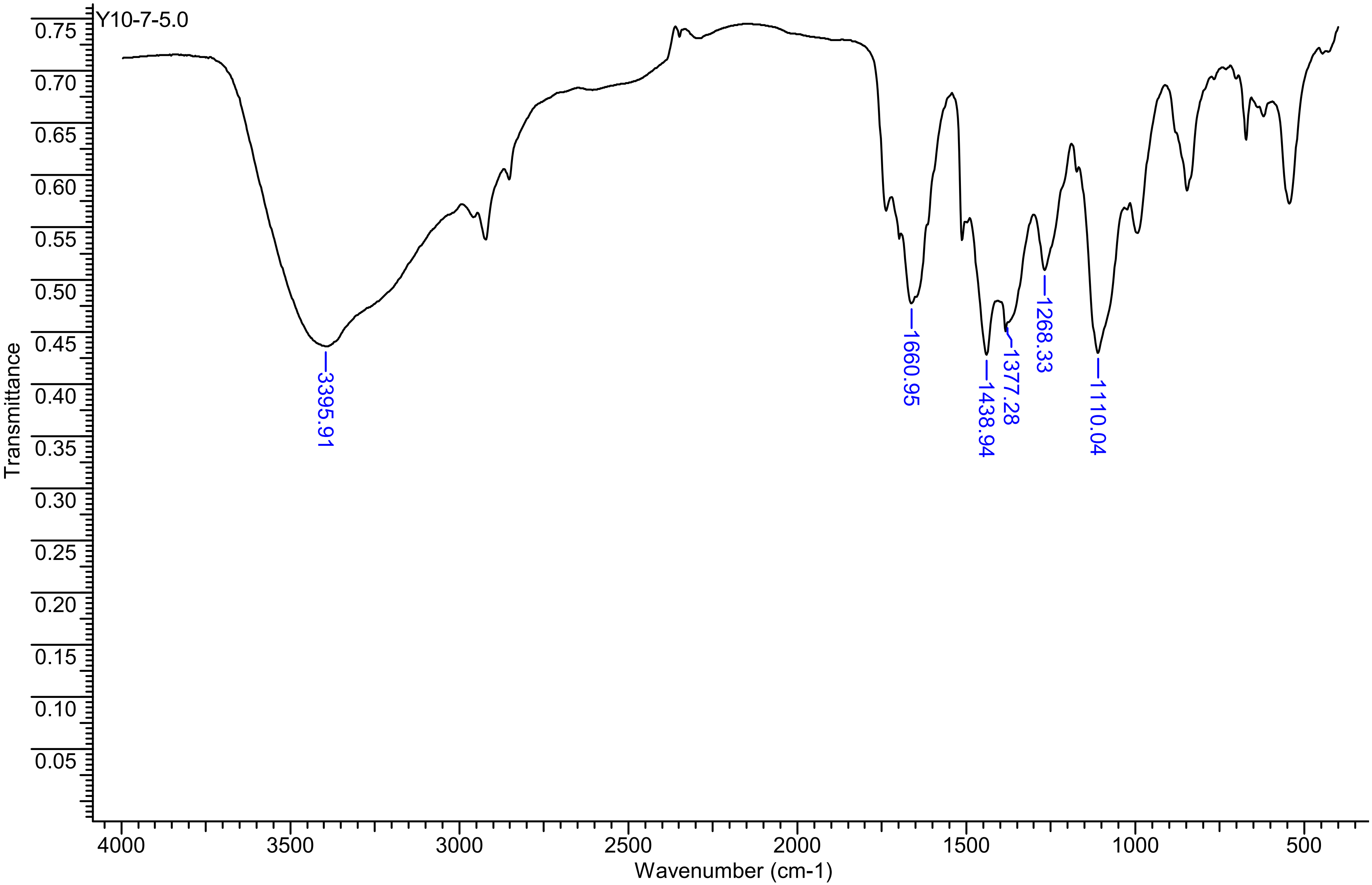

Figure S 28. IR spectrum of compound **4.**

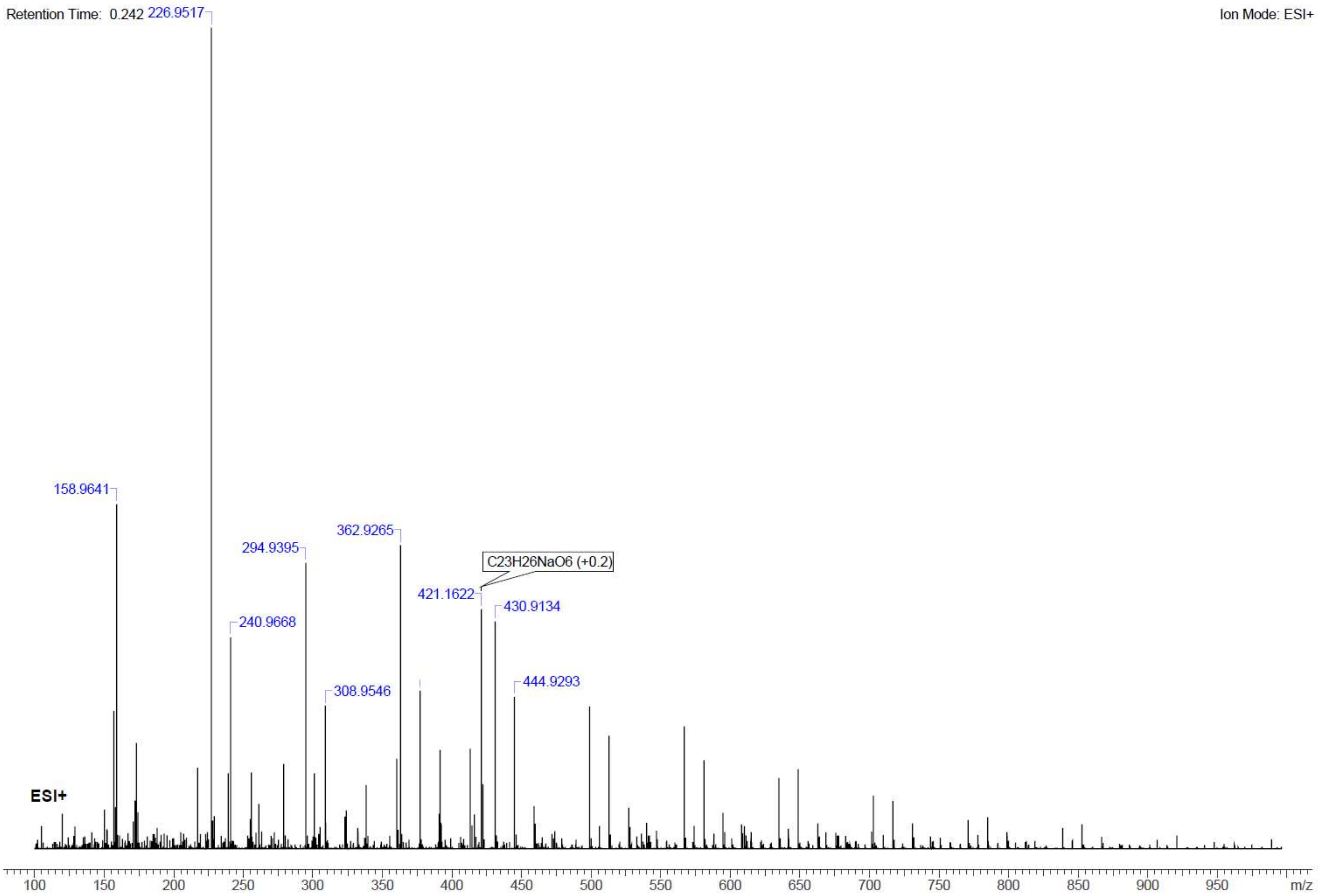

Figure S 29. HR-ESI-MS spectra of compound **4.**

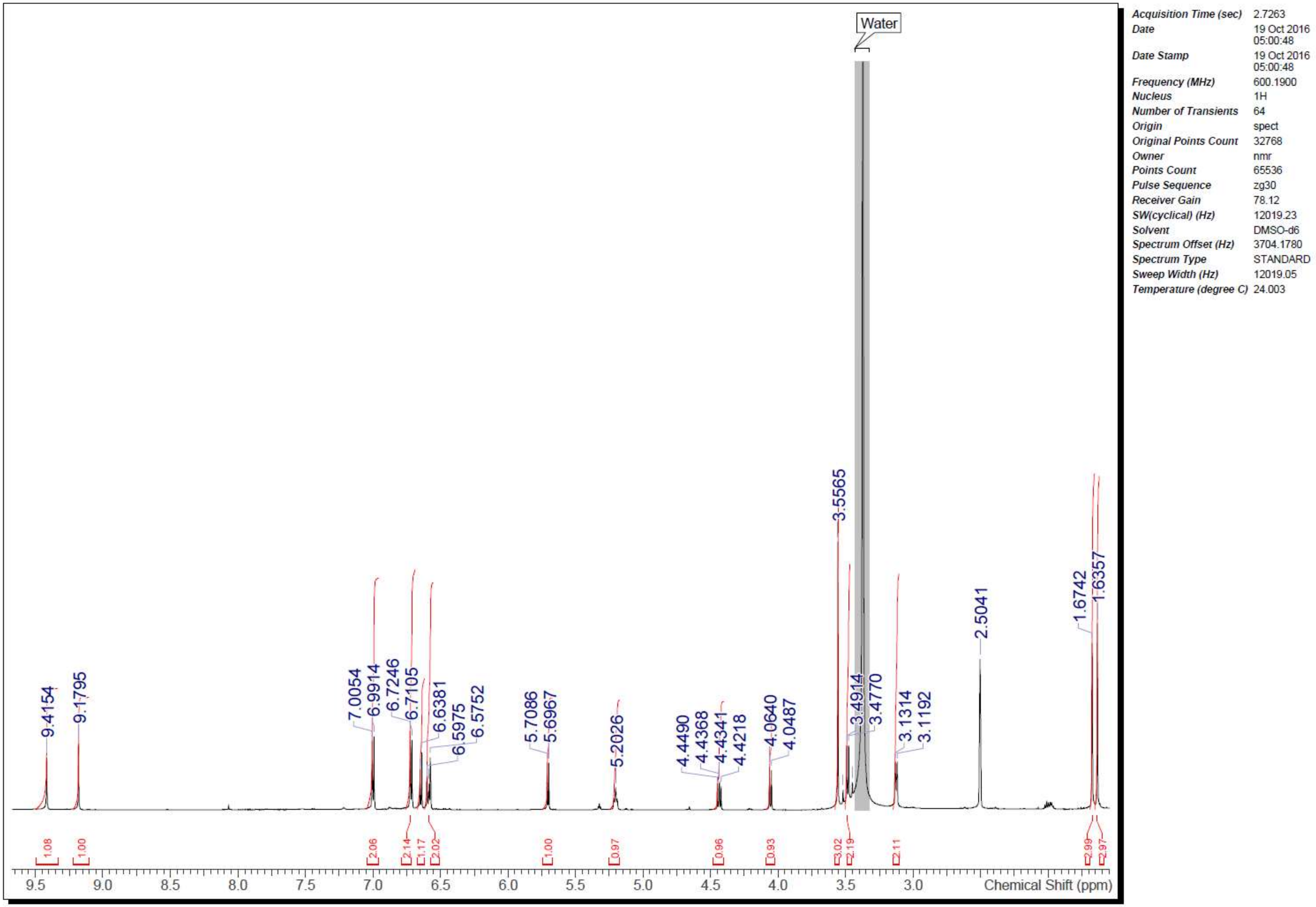

Figure S 30. ¹H-NMR (600 MHz, DMSO-d_6) of compound **4.**

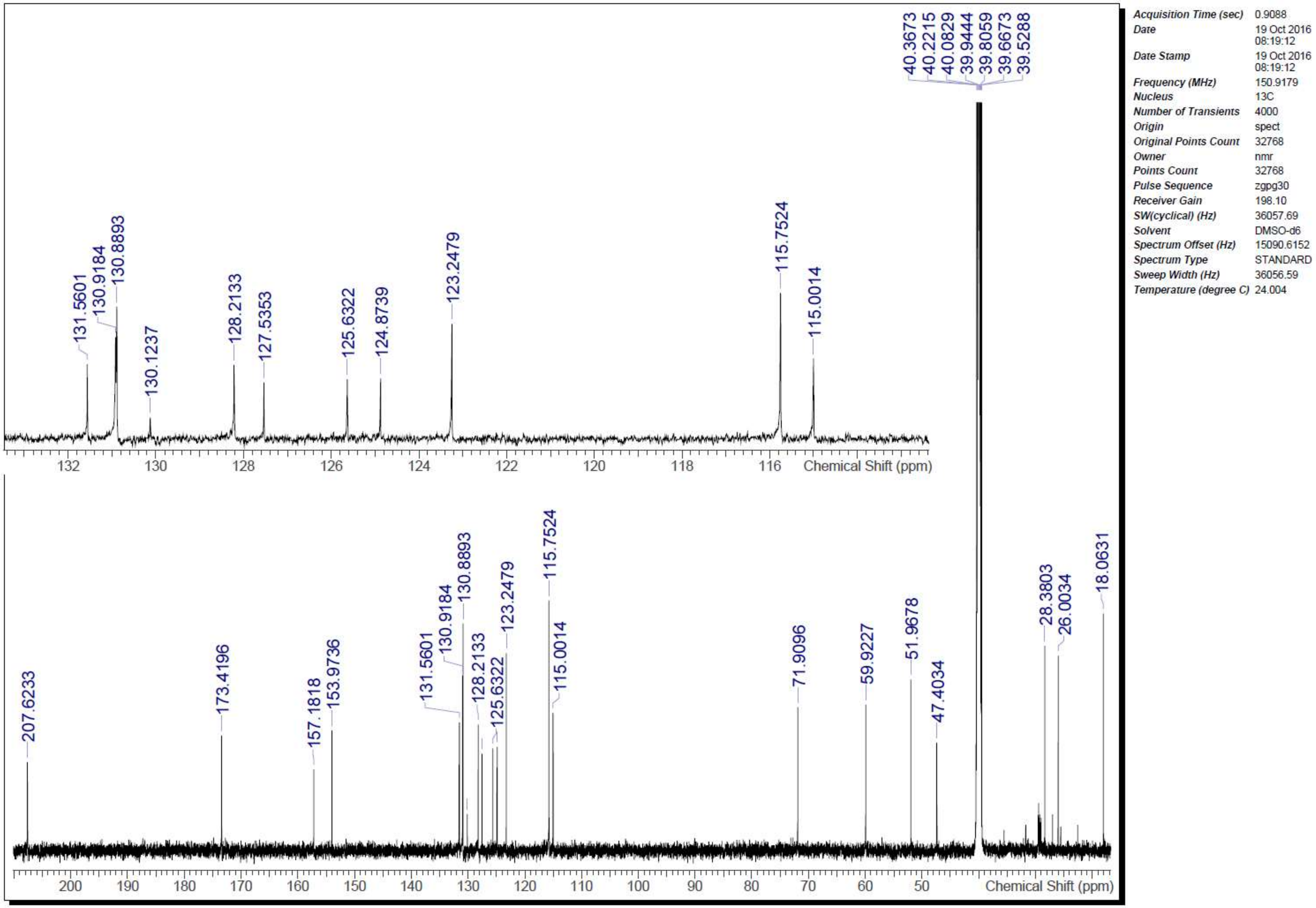

Figure S 31. ^{13}C-NMR (150 MHz, DMSO-d_6) of compound **4.**

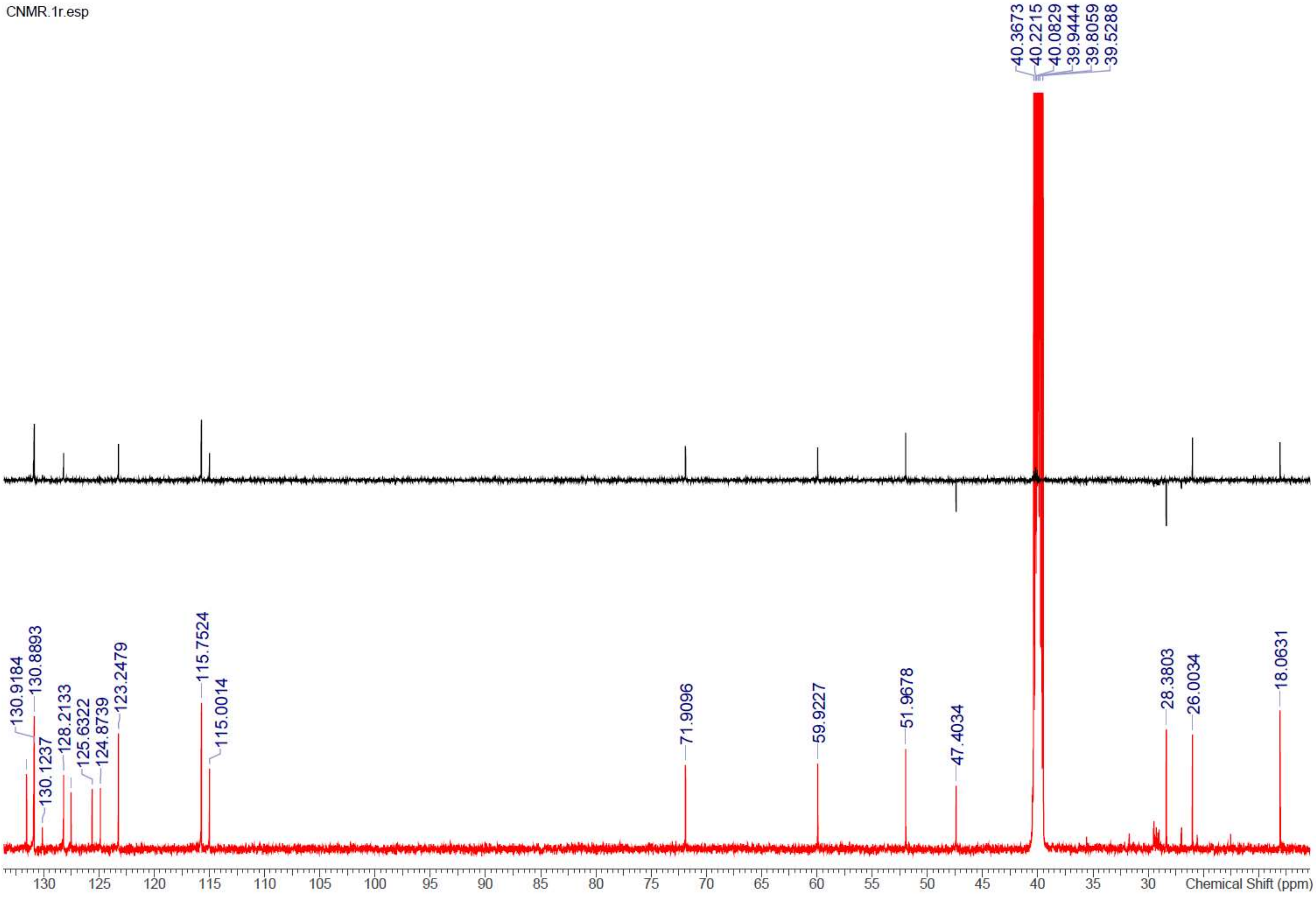

Figure S 32. DEPT of compound **4.**

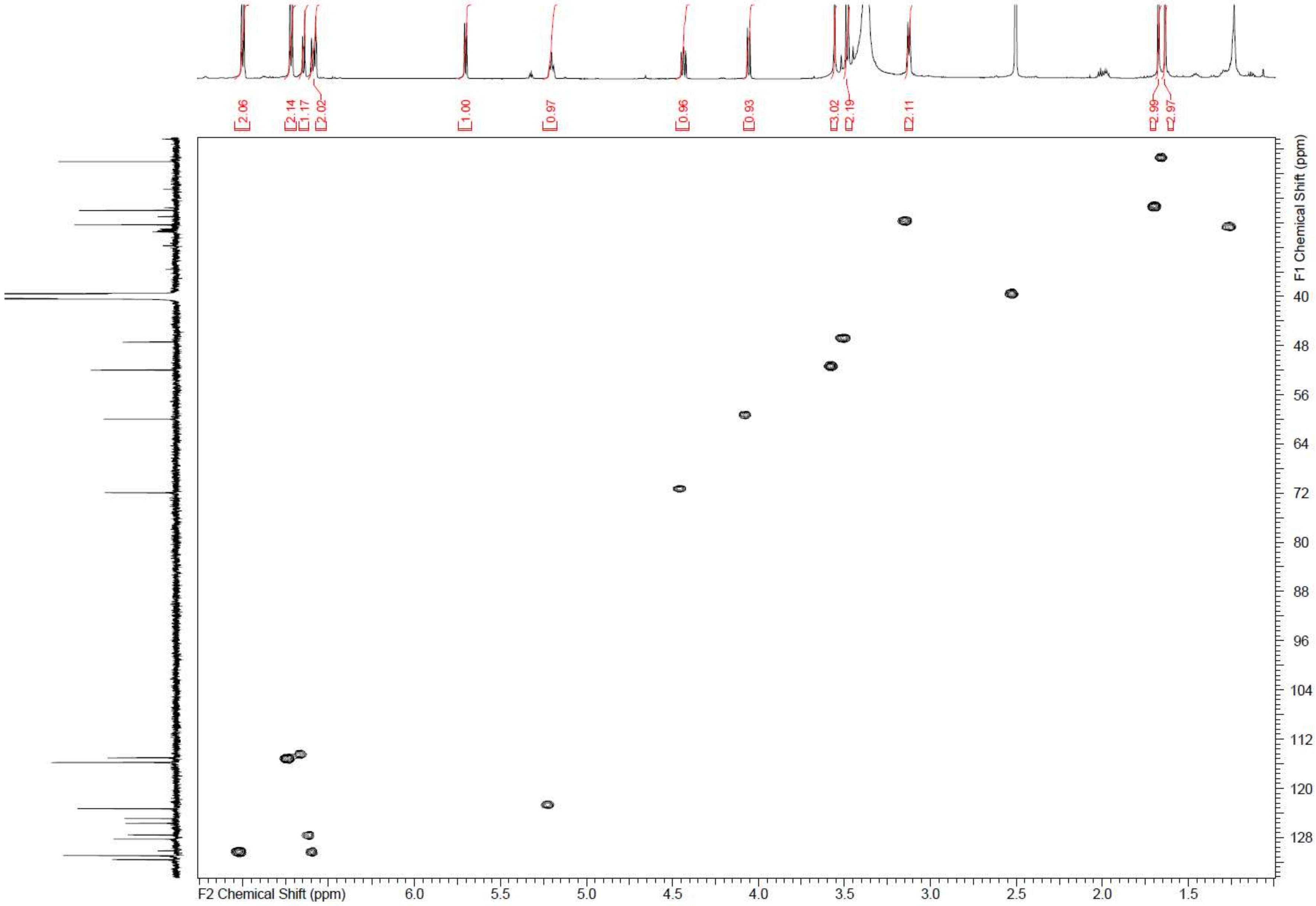

Figure S 33. HSQC of compound **4.**

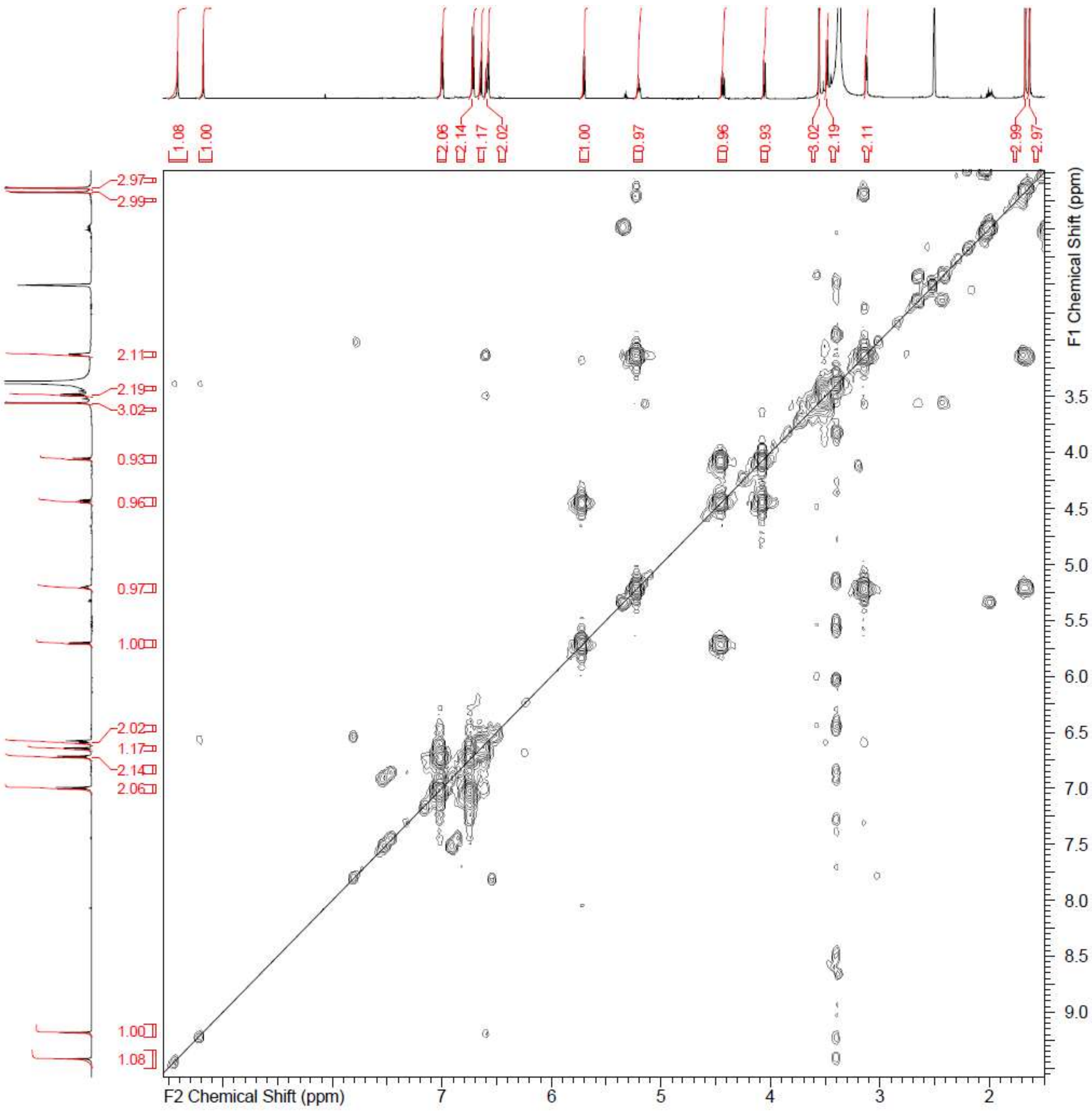

Acquisition Time (sec)	(0.0852, 0.0214)
Comment	5 mm PABBO BB/19F-1H/D Z-GRD Z114607/0133
Date	03 Nov 2016 03:46:48
File Name	C:\Users\Administrator\Desktop\yanxia-20161103\Y10-7-5\3\pdata\1\2rr
Frequency (MHz)	(600.1936, 600.1936)
Nucleus	(1H, 1H)
Number of Transients	16
Origin	spect
Original Points Count	(1024, 256)
Owner	nmr
Points Count	(2048, 1024)
Pulse Sequence	cosygpmfqf
Solvent	DMSO-d6
Spectrum Type	COSY
Sweep Width (Hz)	(12013.36, 11978.70)
Temperature (degree C)	24.008
Title	

Figure S 34. ^{1}H-^{1}H COSY of compound **4.**

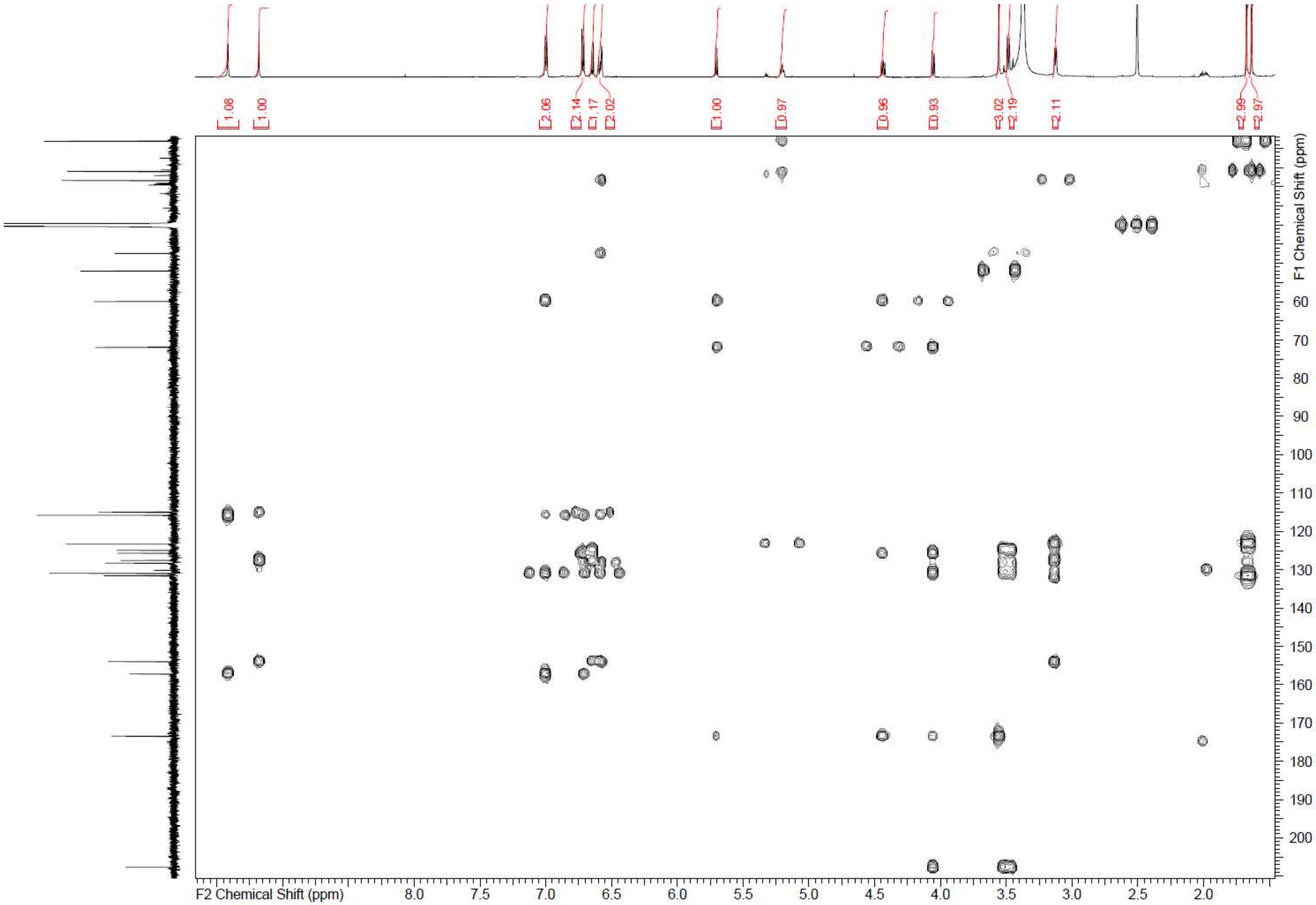

Figure S 35. HMBC of compound **4.**

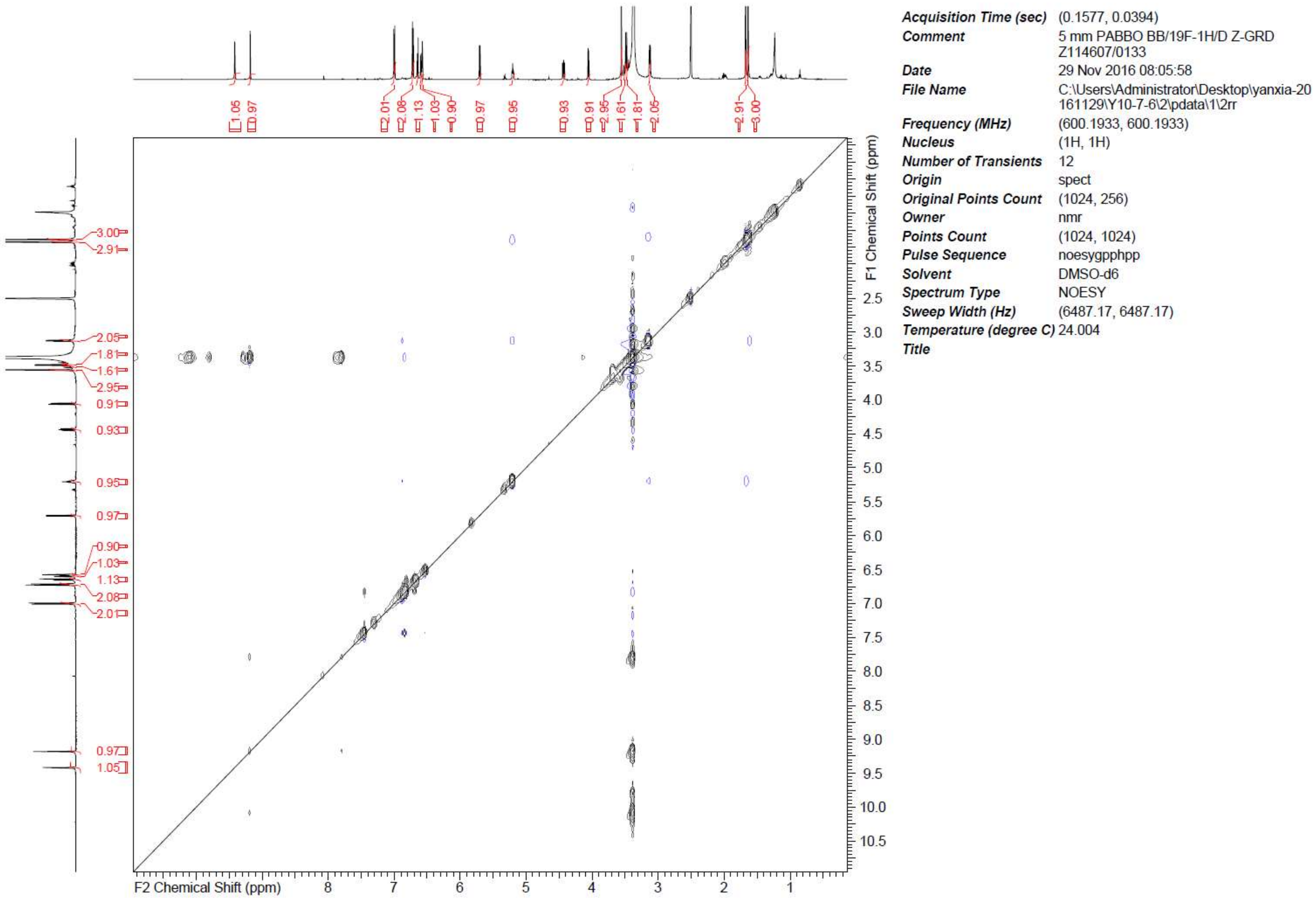

Figure S 36. NOESY of compound **4.**
